# Subarachnoid hemorrhage distinctively disrupts the glymphatic and meningeal lymphatic systems in beagles

**DOI:** 10.7150/thno.100982

**Published:** 2024-09-16

**Authors:** Jiaqi Wang, Tao Lv, Feng Jia, Yang Li, Weiwei Ma, Zhi-Peng Xiao, Weifeng Yu, Heng Zhao, Xiaohua Zhang, Qin Hu

**Affiliations:** 1 Department of Neurosurgery, Ren Ji Hospital, Shanghai Jiao Tong University School of Medicine, Shanghai, China.; 2 Key Laboratory of Anesthesiology (Shanghai Jiao Tong University), Ministry of Education, China.; 3 Department of Radiology, Ren Ji Hospital, Shanghai Jiao Tong University School of Medicine, Shanghai, China.; 4 Jiangxi Key Laboratory of Neurological Diseases, Department of Neurosurgery, The First Affiliated Hospital, Jiangxi Medical College, Nanchang University, Nanchang, Jiangxi, China.; 5 Beijing Institute of Brain Disorders, Laboratory of Brain Disorders, Ministry of Science and Technology, Joint Innovation Center for Brain Disorders, Capital Medical University, Beijing, China.; 6 Loma Linda University School of Medicine, Loma Linda, CA 92350, USA.

**Keywords:** subarachnoid hemorrhage, meningeal lymphatic system, glymphatic system, external CSF drain, beagles

## Abstract

Subarachnoid hemorrhage (SAH) induced acute impairment of the glymphatic system, but few have investigated the dysfunction of the meningeal lymphatic system and their contribution to the pathophysiology of SAH. In addition, most studies were conducted in rodent animals. We aimed to investigate the impact of SAH on glymphatic and meningeal lymphatic function in a large animal model using beagles and to evaluate the effects of intermittent cistern magna CSF drainage on these systems.

**Methods:** The SAH model was created in beagles via endovascular perforation using a digital subtraction angiography machine. Intermittent cistern magna CSF drain was performed daily from 1 d to 3 d after SAH. We examined CSF pressure, neuronal death, enlargement of perivascular space (PVS), hydrocephalus, and neurological and cognitive deficits before and after SAH. The dynamics of glymphatic and meningeal lymphatic functions were analyzed by quantifying the signal intensity of dimeglumine gadopentetate (Gd-DTPA) using T1-weighted magnetic resonance imaging (MRI). Measurements were taken before SAH and at 1 h, 1 week, and 2 weeks post-SAH.

**Results:** SAH in beagles caused significant blood clots, neuronal death, increased CSF pressure, hydrocephalus, and neurological and cognitive deficits. MRI revealed dilated ventricles and enlarged PVS post-SAH. The glymphatic system's function, assessed by Gd-DTPA distribution, showed reduced CSF influx and glymphatic impairment after SAH, particularly in the ipsilateral hemisphere, persisting for a week with partial recovery at 2 weeks. For lymphatic clearance, Gd-DTPA rapidly filled the olfactory bulbs, optic nerves, facial and vestibulocochlear nerves, and spinal nerves under normal conditions. SAH caused delayed and reduced Gd-DTPA efflux outflow in these areas, disrupting lymphatic clearance. Despite initial dysfunction, increased hemoglobin levels in cervical lymph nodes indicated active blood clearance post-SAH, with recovery by 2 weeks. Treatment with intermittent cistern magna CSF drain significantly ameliorated the glymphatic and meningeal lymphatic dysfunction after SAH.

**Conclusion:** SAH impaired both glymphatic and meningeal lymphatic functions in beagles, with better restoration of lymphatic function post-SAH, which may contribute to functional recovery after SAH. External CSF drain is an effective therapeutic approach to facilitate the recovery of glymphatic and meningeal lymphatic function following SAH.

## Introduction

Subarachnoid hemorrhage (SAH) is a devastating neurological condition that leads to significant morbidity and mortality. Understanding the mechanisms underlying brain injury and recovery after SAH is crucial for developing effective therapeutic strategies 1. Recent studies have highlighted the importance of cerebrospinal fluid (CSF) clearance pathways, particularly the glymphatic and meningeal lymphatic systems, in maintaining brain health and facilitating recovery after brain injuries 2,3. Nevertheless, how these two systems are implicated in SAH remains unknown.

The glymphatic system is a highly specialized network responsible for the clearance of metabolic waste and solutes from the brain. It operates through the movement of CSF along perivascular spaces (PVS), driven by arterial pulsations and facilitated by aquaporin-4 channels on astrocytic endfeet 4. This system allows CSF to enter the brain parenchyma, mix with interstitial fluid, and then drain along the perivenous spaces. Imaging studies using contrast agents such as dimeglumine gadopentetate (Gd-DTPA) can track the distribution of CSF within the brain parenchyma and PVS using T1-weighted magnetic resonance imaging (MRI), providing insights into the function of the glymphatic system 5.

In contrast, the meningeal lymphatic system comprises lymphatic vessels located along the dura mater and associated with cranial and spinal nerves 6. This system plays a critical role in draining CSF and associated waste products from the subarachnoid space to the peripheral lymphatic system 7,8. The meningeal lymphatic vessels are primarily located around the dural venous sinuses and along cranial nerves such as the olfactory bulbs, optic nerves, facial and vestibulocochlear nerves, and spinal nerves 9,10. The drainage process can be visualized by monitoring the signal intensity (SI) of contrast agents in these regions using MRI 11.

The glymphatic and meningeal lymphatic systems are interconnected and collaboratively maintain brain homeostasis by clearing waste 12. As we have discussed, the glymphatic system facilitates the CSF flow through the brain parenchyma via periarteriolar spaces, and this waste-laden CSF then exits through perivenous spaces and enters the meningeal lymphatic vessels, which are located along the venous sinuses in the dorsal brain and cranial nerves in the ventral brain. These lymphatic vessels then drain the CSF and its waste products to peripheral lymph nodes, completing the clearance process. While dysfunction of the glymphatic system has been well-documented in the early stages of SAH in rodents 13-15, few studies have investigated the dysfunction of the meningeal lymphatic system or compared the contributions of both systems to brain damage after SAH, especially in large animal models.

In this study, we established an SAH model with endovascular perforation in beagles to examine SAH-induced brain damage. Given that the canine brain closely resembles the human brain in terms of neuroanatomy and physiology, our results provide a more accurate simulation of the pathophysiology of SAH in humans. We aimed to analyze the dynamics of the glymphatic and meningeal lymphatic systems by quantifying the SI of Gd-DTPA in various brain and lymphatic regions at defined time points post-SAH. In addition, we investigated the therapeutic effect of intermittent cistern magna CSF drain on the two systems. The dynamics of the glymphatic system were assessed by tracking the distribution of Gd-DTPA in the brain parenchyma and perivascular spaces. On the other hand, the meningeal lymphatic system was evaluated by monitoring the SI in the perineurium of cranial and spinal nerves, the dural venous sinus, and cervical lymph nodes. Our findings demonstrate the distinct roles and behaviors of the glymphatic and meningeal lymphatic systems in CSF clearance post-SAH, paving the way for developing targeted therapies to improve outcomes for SAH patients.

## Methods and Materials

### Experimental design and ethical approval

Fourteen male beagles, aged 10-12 months and weighing 8.84 ± 0.33 kg, were randomly divided into 4 groups: Sham (3), SAH-1d (3), SAH-2w (4) and SAH + CSF drain (4). The experimental design and timeframes were depicted in Figure S1A. The beagles were purchased from Shanghai Jiao Tong University Agricultural Experimental Animal Farm Co., Ltd (Shanghai, China). They were housed individually in cages, with average room temperature, humidity and a regular circadian rhythm. All methods were performed according to National Institutes of Health guidelines and approved by the Ethics Committee of Shanghai Jiao Tong University Agricultural Experimental Animal Farm Co., Ltd (approval No. JDLL20230610).

### Animal anesthesia

Anesthesia was performed before MRI scan and SAH operation. The beagles were anesthetized with Tiletamine Hydrochloride and Zolazepam Hydrochloride (Zoletil®50, Virbac, France) (10 mg/kg, intramuscularly) combined with Xylazine Hydrochloride (Baite®, Best Technology, Changsha, China) (0.01 mL/kg) 16. Atropine sulfate (Quanyu Biotechnology, Shanghai, China) (0.05 mg/kg) was injected subcutaneously 15 min before anesthesia. Tracheal intubation was performed to maintain a patent airway. Body temperature, blood pressure, heart rate, and respiratory rate were continuously monitored. The maintenance dose for anesthesia was 1/3 of the initial, and given every 40 min. Meloxicam (Meikang®, Baoding Sunshine Herbal Medicine Co., Ltd., Hebei, China) (0.2 mg/kg, subcutaneously) was used for pain relief.

### Intracisternal injection of contrast media

Gd-DTPA (Bei Lu Pharmaceutical Co., Ltd., Beijing, China) was injected into the cistern magna to trace CSF circulation as previously reported 17. Beagles were anesthetized, placed in the prone position and their heads were attached to a stereotaxic frame (RWD co., Ltd., Shenzhen, China). After shaving the suboccipital area and sterilizing the scalp with 75% ethyl alcohol, a 0.5 cm midline incision was made below the occipital protuberance. The subcutaneous tissue and neck muscles were carefully separated. A 22-Gauge venous indwelling needle (Linhwa®, Suzhou, China) was used to puncture the cisterna magna membrane between the occipital protuberance and the atlas at 45-degree angle. The outflow of CSF confirmed successful puncture. After collecting 0.3 mL CSF, 0.3 mL Gd-DTPA (0.5 g/mL) was injected into the cistern magna through the needle at the rate of 0.15 mL/min. The needle was held in place for 3 min before gently removed. The skin incision was then carefully sewn up, disinfected and covered with sterile dressing.

### CSF pressure measuring

A subdural catheter and a pressure transducer (Codman, Johnson & Johnson, USA) were used for CSF pressure measuring at the cisterna magna after anesthesia 18. The catheter was positioned in the SAS, and CSF pressure was recorded.

### SAH model

SAH is induced in beagles via endovascular perforation with a digital subtraction angiography machine (Artis Q Zeego, Siemens Healthineers, Erlangen, Germany). The beagles were fasted for at least 8 h prior to the procedure. After anesthesia, the beagles were placed in a supine position with the head firmly fixed in the stereotactic head holder. Both the navigating microcatheters and the microguidewires were heparinized. The right femoral artery was carefully exposed and then punctured using a modified Seldinger approach. A 5F introducer sheath (5F, 10 cm, Terumo, Tokyo, Japan) was then positioned into the right femoral artery and secured. To prevent blood coagulation, 2500 IU of unfractionated heparin was administered through the sheath. Under X-ray fluoroscopy, a 4F guiding catheter (100 cm, Vertebra, Cordis Corporation, Hialeah, Florida, USA) was inserted and navigated into the aortic arch, then advanced further into the common cervical arteries and the vertebral arteries. Iomeprol (Bracco, Milan, Italia) was injected through the guiding catheter for angiography to evaluate the course of the bilateral internal cervical arteries and vertebral arteries. Due to the tortuous nature of the internal carotid arteries in beagles we selected the vertebral arteries as the pathway to reach the Circle of Willis. A 0.014-inch slightly-shaped super-slip microguidewire (Synchro-14, 200 cm, Stryker Corporation, Kalamazoo, Michigan, USA) along with a microcatheter (Echelon-10, 150 cm, Medtronic Plc, Minneapolis, Minnesota, USA) was introduced into the Circle of Willis through the guiding catheter. An endovascular puncture was performed at the right posterior communicating artery using the tip of the microguidewire. The extravascular outflow of Iomeprol confirmed the successful induction of SAH. Ten seconds after the puncture, the microguidewire was withdrawn, followed by the microcatheter, the guiding catheter, and finally the introducer sheath. The right femoral artery was then ligated, and the incision was sutured. The trachea was extubated and the beagle was placed in the recovery cage under a heating lamp until fully awake. The sham-operated animal received all the surgery performance but not the insertion of the catheters and the microguidewires.

### Intermittent cistern magna CSF drain

To investigate whether external CSF drain benefits the recovery of the glymphatic and meningeal lymphatic function, intermittent cistern magna CSF drain was performed using a 22-Gauge venous indwelling needle, as described above, at 1 d, 2 d and 3 d after SAH. The first drain was performed after the 12-h MRI scan, approximately 13 h post SAH. Each time, 2 ml of CSF was slowly drained while monitoring CSF pressure. Body temperature, blood pressure, heart rate and respiratory rate were monitored for at least 3 hours in the recovery cage.

### MRI protocol

The MRI was done with a 3.0-Tesla MRI scanner (Signa HDXT, GE Healthcare, Milwaukee, Wisconsin, USA). Sagittal-, coronal- and transverse-plane images were obtained with a 3D T1-weighted sequence--magnetization prepared rapid gradient-echo (MP-RAGE). Parameters for scanning were set as followed: Bw = 31.2 kHz, TE = 2.1 ms, TR = 6.6 ms, TI = 0 ms, flip angle = 15°, resolution = 320 × 256, slice thickness = 3 mm, Gap = 0, Fov = 20 × 20, Nex = 1. Baseline images were obtained during each series of MRI scans before injection of Gd-DTPA (0 h). Enhanced images were acquired at 0.5 h, 1 h, 2 h, 3 h, 4 h, 6 h, 8 h, 10 h, and 12 h after Gd-DTPA injection. MRI images were analyzed with RadiAnt DICOM Viewer (Medixant, Poznan, Poland). Regions of interest were respectively depicted in corresponding figures. SI fold change was calculated as *SI fold change = (SI- SI_baseline_)/SI_baseline_* as previously reported 5.

### Evaluation of hydrocephalus

Using RadiAnt DICOM Viewer, volumes of lateral ventricles were calculated by summing up sectional areas in all coronal-plane images, multiplied by the slice thickness (3 mm). Evan's index was used to evaluate the degree of hydrocephalus 19, calculating as the ratio of the maximal width between bilateral frontal horns of the lateral ventricles to the maximal internal diameter of the skull at the same level in transverse-plane MRI images.

### Neurological scoring

The Neurological Deficit Score (NDS) was used to evaluate neurological deficits following SAH 20. Scores range from 0 to 100, based on evaluation of consciousness level, respiration, cranial nerve functions, reflex, motor, sensory as well as behavior.

### Cognitive tests

To evaluate the cognitive ability of the beagles, spatial working memory and problem-solving ability were assessed as previously reported 21. Beagles were fasted for at least 6 h prior to the tests. For the spatial working memory test, the beagles were placed in a cage at the center of the study room. The food reward (a small piece of ham) was shown to the beagles and then placed in one corner of the room. The beagles were then taken out of the study room for 30 s before being reintroduced to the center of the room to search for the food reward. The performance was scoreed as follows: 1, goes directly towards the food; 2, finds the food within 1 min; 3, searches for the food but does not find it within 1 min; 4, makes no attempt to search for the food.

For the problem-solving ability test, the food reward was covered by a transparent plastic box and put in front of the beagles. The scoring was as follows: 1, obtains the food within 2 min; 2, attempts to get the food but does not obtain all of it within 2 minutes; 3, sniffs the box but does not try to retrieve the food; 4, makes no attempt to get the food. Each test was repeated three times, and the total score from the both tests was used to evaluate the cognitive ability of the beagles.

### SAH grading

The beagles were euthanized via overdose anesthesia. After transcardially perfused with ice-cold normal saline (0.9% NaCl solution), the brains were extracted. The severity of SAH was assessed by the Sugawara SAH grading system 22. In detail, the basal cistern was divided into 6 segments, with each given a grade according to the content of blood: 0, no subarachnoid blood; 1, minimal subarachnoid blood; 2, moderate blood clot with recognizable arteries; 3, blood clots obliterating all arteries. A total score ranging from 0 to 18 was finally achieved by summing up the 6 segments.

### Histological experiments

The brain was fixed by 10% formaldehyde for 48 h, rinsed with 0.01 M phosphate buffered saline (PBS) and dehydrated through graded ethanol series starting with 30% and ending with 100%. Xylene was used to clear the tissue. The brain was embedded in paraffin and then sectioned on a microtome into 5 µm thick sections. After deparaffinization, hematoxylin-eosin and Nissl staining were performed using Hematoxylin and Eosin Staining Kit (Beyotime, Shanghai, China) and Nissl Staining Solution (Beyotime, Shanghai, China). Images were captured using a digital pathology slide scanner (KF-PRO-120, KFBIO, Ningbo, China). Vascular and perivascular areas were measured using Image J Software (1.54j, NIH, USA).

### Hemoglobin content detection

Cervical lymph nodes and sacral lymph nodes were isolated from Sham, SAH-1d and SAH-2w beagles after euthanasia. The lymph nodes were homogenized with 0.01 M PBS (1 mL per 100 mg tissue), centrifuged at 10,000 g for 10 min, and the supernatants were collected. The hemoglobin content was detected with the Hemoglobin Colorimetric Assay Kit (Beyotime, Shanghai, China) according to the manufacturer's instrument. For the standard curve, 0.5 μL, 1 μL, 2 μL, 4 μL and 8 μL beagle's blood was diluted in 1 mL PBS. After incubation for 1 h, the absorbance at 410 nm was measured using a spectrophotometer (LB 942, Berthold Technologies, Germany).

### Statistical analysis

All statistical analyses were performed using Graphpad Prism 9.5 (GraphPad Software, Boston, Massachusetts, USA) and SPSS26.0 (IBM®, New York, USA). The results were expressed as mean ± SD. Data normality was tested using the Shapiro-Wilk Test. Statistical significance between 2 groups was analyzed using the Student *t* test; for multiple groups comparations, One-way ANOVA followed by a Tukey test (normally distributed) or a Dunn's test (nonnormally distributed) was used.

## Results

### SAH induced brain damage in beagles

**Figure 1:**
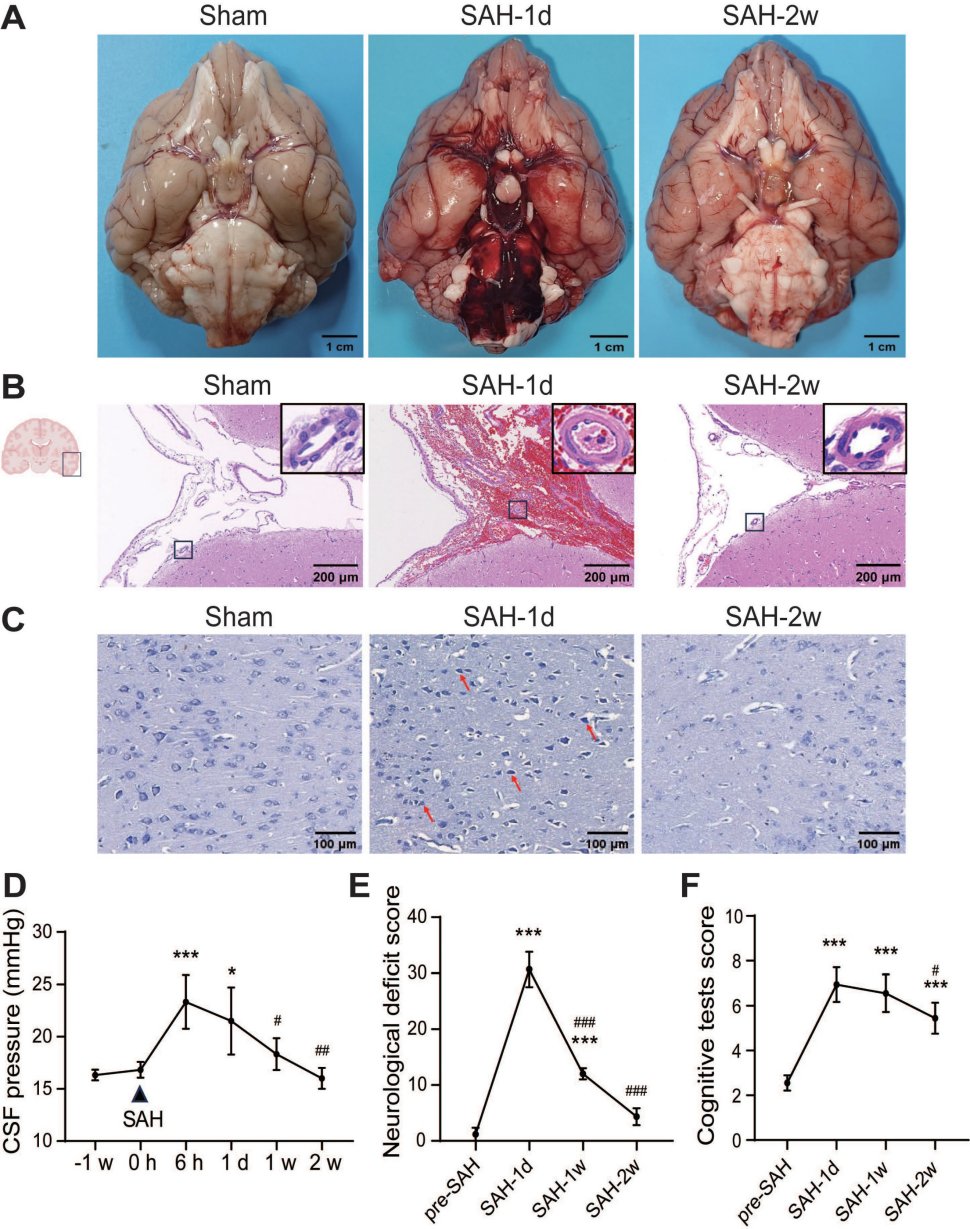
**SAH caused early brain injury and long-term neurological and cognitive deficits in beagles.** (A) Macroscopic view of blood clots at the base of the brain. No visible blood clots were observed at the ventral brain surface in the Sham group; lots of blood clots were observed at the ventral brain surface 1 day after SAH and mostly absorbed at 2 weeks. (B) Representative images of hematoxylin-eosin staining of brain sections. After SAH, the SAS was filled with erythrocytes. The arteries were wall thickening, the lumen was narrowing, and endothelial cells were shrunken. The erythrocytes in the SAS were remarkably reduced and the vasospasm was mitigated 2 weeks later. (C) Representative images of Nissl staining in the temporal cortex. In sham animals, neurons are arranged in polarization, and presented with abundant cytoplasm and clear nuclei. 1 day after SAH, neurons lost the polarization and shrunk with dark nuclei (red arrows). The morphology of neurons improved with round cell body and evenly distributed Nissl particles, however, the cell density decreased 2 weeks after SAH. (D) The alteration of CSF pressure before SAH and 6 h, 1 d, 1 week and 2 weeks after SAH. SAH increased CSF pressure 6 h after SAH, stayed high at 1 d, and dropped to normal level at 1 week. (E) SAH induced severe neurological deficits 24 h after SAH and had a spontaneous improvement at 1 week and further improved at 2 weeks. (F) SAH induced severe cognitive deficits 24 h after SAH, and improved at 2 weeks. One-way ANOVA, n = 3 or 6; * P < 0.05, *** P < 0.001, compared to 0 h or pre-SAH; # P < 0.05, ## P < 0.01, ### P < 0.001, compared to 6 h or SAH-1d. CSF: cerebrospinal fluid; SAS: subarachnoid space; SAH: subarachnoid hemorrhage.

The SAH model was established in beagles by puncturing the Circle of Willis with a microguidewire (Figure S1B-C). In sham-operated beagles, no blood clots were observed at the base of the brain, while SAH caused significant blood clots around the Circle of Willis and the ventral brain stem, which disappeared by 2 weeks (Figure 1A). The SAH score dropped from 12 ± 0.8 at 1 day to 0.67 ± 0.47 at 2 weeks (Figure S1D). Two beagles died shortly after SAH, and the mortality was 18.18% (2 out of 11 animals). Hematoxylin-eosin staining showed erythrocyte aggregation in the SAS 1 day after SAH, with artery wall thickening, lumen narrowing, and shrunken endothelial cells, which improved by 2 weeks (Figure 1B). SAH induced neuronal death in the temporal cortex, with shrunken cell bodies and darker nuclei, which improved at 2 weeks (Figure 1C). CSF pressure increased from 16.8 ± 0.7 mmHg to a peak of 23.3 ± 2.4 mmHg at 6 h, remained high until 1-day post-SAH, and normalized at 1 week (Figure 1D). SAH beagles showed severe neurological deficits and cognitive decline at 1 day, with neurological improvements at 1 week and near recovery by 2 weeks, while cognitive recovery began at 2 weeks (Figure 1E-F).

**Figure 2:**
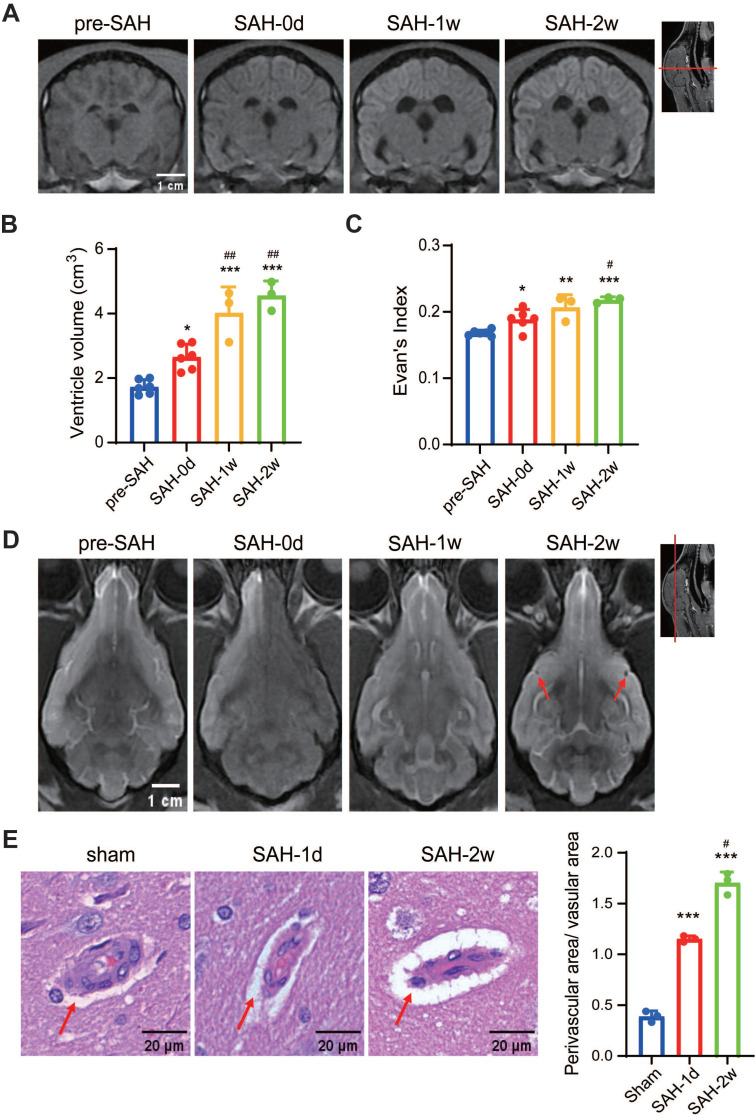
** SAH induced hydrocephalus and enlargement of PVS in beagles.** (A) Representative coronal-plane MRI images showing enlargement of the lateral ventricles after SAH. (B) SAH induced enlargement of lateral ventricles within hours after SAH, and worsened at 2 weeks. (C) Quantification of the Evan's index before, 1 h, 1 week and 2 weeks after SAH. (D) Representative transverse-plane MRI images showing enlargement of PVS (red arrows) 2 weeks after SAH. Images were taken 12 h after Gd-DTPA injection. (E) Left, representative hematoxylin-eosin staining showing the progressive enlargement of PVS (red arrows) after SAH; Right, statistic of the Left images. One-way ANOVA, n = 3 or 6; * P < 0.05, ** P < 0.01, *** P < 0.001, compared to pre-SAH or Sham; # P < 0.05, ## P < 0.01, compared to SAH-0d or SAH-1d. PVS: perivascular space; SAH: subarachnoid hemorrhage.

In pre-SAH beagles, MRI images showed well-defined brain cortex and subcortical structures, normal-sized lateral and third ventricles, and closely arranged sulci and gyri (Figure 2A). After SAH, acute hydrocephalus developed within hours, with dilated lateral ventricles and temporal horns, which progressively enlarged over 2 weeks (Figure 2A-B). Evan's index increased significantly 1-week post-SAH and remained high for 2 weeks (Figure 2C). No enlarged PVS was seen in pre-SAH animals, but it was notable in SAH beagles at 2 weeks (Figure 2D), confirmed by hematoxylin-eosin staining (Figure 2E). These findings indicated that SAH induced acute neuronal death, elevated CSF pressure, hydrocephalus, long-term glymphatic dysfunction, and neurological and cognitive deficits in beagles.

### Assessment of glymphatic system function under normal conditions using Gd-DTPA in beagles

**Figure 3:**
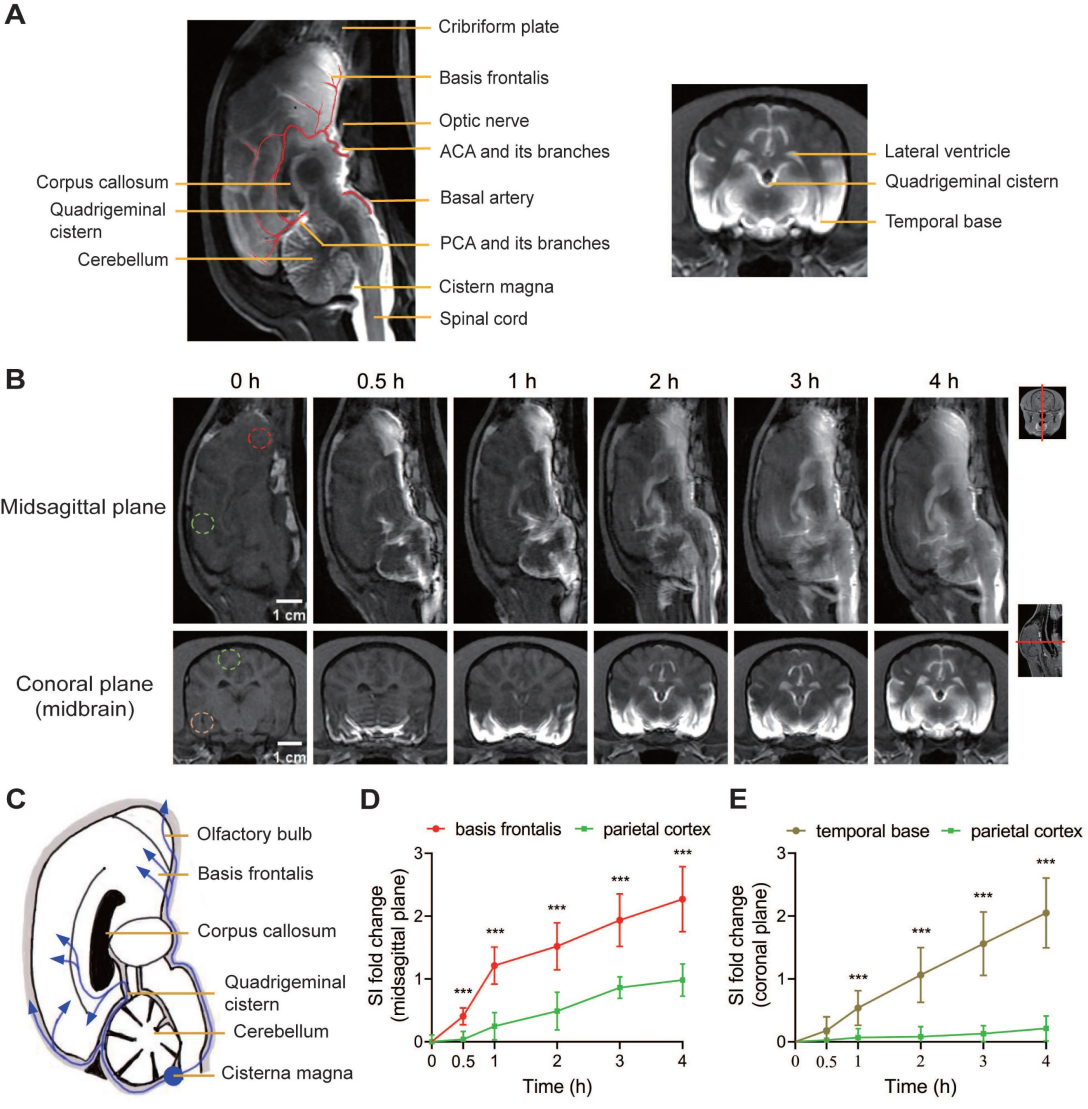
** MRI-based evaluation of glymphatic function in pre-SAH beagles.** (A) Anatomical diagrams of MRI images showing the major arteries (ACA and PCA and their branches), lateral ventricles, and quadrigeminal cistern. Key structures labeled include the cribriform plate, basis frontalis, optic nerve, corpus callosum, quadrigeminal cistern, cerebellum, cisterna magna, and spinal cord. (B) Representative MRI images showing the progressive penetration of Gd-DTPA in the brain of sham beagles at various time points after Gd-DTPA injection. After injection into the cisterna magna, Gd-DTPA began to diffuse from the ventral side of the brain, penetrated into the brain parenchyma alongside the major arteries, and reached the cerebellum and ventricles. (C) Schematic showing the cerebrospinal fluid diffusion routes from the cisterna magna through the brain structures, highlighting the pathways through the basis frontalis, olfactory bulb, quadrigeminal cistern, corpus callosum, and cerebellum. (D) Quantification of SI fold changes over time in the basis frontalis and parietal cortex. E) Quantification of SI fold changes over time in the temporal bases and parietal cortex. Parametric paired t-test, n = 9; *** P < 0.001, compared to the parietal cortex. ACA: anterior cerebral artery; MRI: magnetic resonance imaging; PCA: posterior cerebral artery; SI: signal intensity.

The function of the glymphatic system was assessed by mapping the enrichment of Gd-DTPA within the brain parenchyma at defined time points after cisterna magna injection in beagles before SAH. In sagittal plane MRI images, key glymphatic flow routes, including the basal artery (BA), anterior cerebral artery (ACA), and posterior cerebral artery (PCA), were visible (Figure 3A). Following injection, Gd-DTPA rapidly entered the basal cisterns, spinal SAS, and PVS of BA, ACA, and PCA within 30 min. At 1 h, it spread along major arteries and their branches, reaching the cerebellum; by 2 h, it expanded into the brain cortex and cerebellar parenchyma through PVS. By 4 h, Gd-DTPA had diffused throughout the brain, showing a clear PVS pattern (Figure 3B, top row). In coronal-plane images, enhancement first appeared around the SAS of basal cisterns and temporal base, then dispersed to the brain parenchyma and ventricular system by 1 to 2 h, and finally filled most of the ventral brain by 3 to 4 h (Figure 3B, bottom row). The accumulation of Gd-DTPA peaked at 4 h, began to dissipate at 6 h, and residual intensity was still visible 12 h after Gd-DTPA injection (Figure S2A-B). The sequential appearance of Gd-DTPA indicated its bulk flow routes, diffusing from the cistern magna through SAS and peri-arterial spaces to various brain regions (Figure 3C). SI changes showed faster and higher diffusion in the basis frontalis and temporal bases compared to the parietal cortex, indicating predominant diffusion into the ventral brain regions (Figure 3D-E). The contrast agent diffused rapidly in the first hour and then slowed down.

### SAH impaired the glymphatic function in beagles

SAH impaired the glymphatic function in beagles, as demonstrated by significant changes in the distribution of Gd-DTPA in both sagittal and coronal sections.

**Figure 4:**
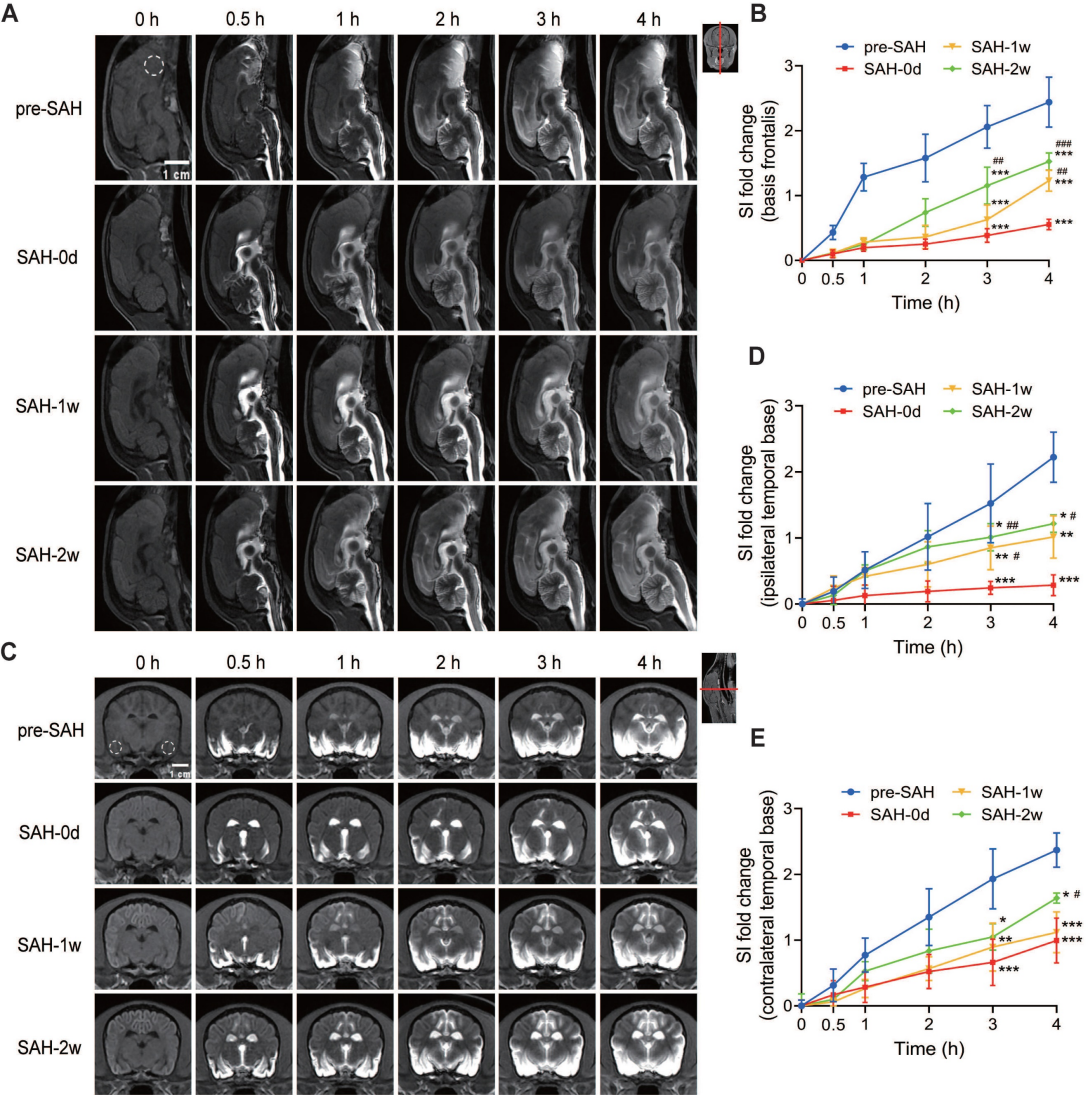
** The glymphatic function was impaired after SAH and partially recovered at 2 weeks.** 0 h represents the baseline; 0.5 h, 1 h, 2 h, 3 h, and 4 h represent time points after Gd-DTPA injection. (A) Representative midsagittal-plane MRI images showing the progressive penetration of Gd-DTPA into the brain parenchyma before SAH (pre-SAH, 1st row), 1 h after SAH (SAH-0d, 2nd row), 1 week after SAH (SAH-1w, 3rd row), and 2 weeks after SAH (SAH-2w, 4th row). (B) Quantification of SI fold changes over time in the basis frontalis. Gd-DTPA parenchymal penetration was significantly impaired within hours after SAH and partially improved at 2 weeks post-SAH. (C) Representative coronal-plane MRI images showing the progressive penetration of Gd-DTPA into the brain parenchyma before SAH (pre-SAH, 1st row), 1 h after SAH (SAH-0d, 2nd row), 1 week after SAH (SAH-1w, 3rd row), and 2 weeks after SAH (SAH-2w, 4th row). (D and E) Quantification of SI fold changes over time in the ipsilateral temporal base (D) and contralateral temporal base (E). Gd-DTPA parenchymal penetration was impaired within hours after SAH and partially improved at 2 weeks. The impairment was more severe on the ipsilateral side. One-way ANOVA, n = 3 or 6; * P < 0.05, ** P < 0.01, *** P < 0.001, compared to pre-SAH; # P < 0.05, ## P < 0.01, ### P < 0.001, compared to SAH-0d. MRI: magnetic resonance imaging; SAH: subarachnoid hemorrhage; SI: signal intensity.

In the midsagittal-plane MRI images (Figure 4A), the progressive penetration of Gd-DTPA into the brain parenchyma was observed before SAH (pre-SAH) and at various time points post-SAH (0 days, 1 week, and 2 weeks). One hour after SAH, there was a significant decrease in Gd-DTPA distribution in the PVS and brain parenchyma. Thirty minutes after Gd-DTPA injection, enhancement was visible in the spinal subarachnoid space (SAS), cerebellum, lateral ventricles, and cerebral fissures, but not in the frontal lobe and ventral brain. Four hours after injection, the diffusion of Gd-DTPA in the arterial PVS and adjacent brain parenchyma was significantly reduced, indicating occlusion of PVS routes following SAH. The late efflux of Gd-DTPA in the brain parenchyma was also compromised after SAH, showing a significant decrease of SI in basis frontalis (Figure S2A and C) from 6 h to 12 h after Gd-DTPA injection. This impairment persisted for 1 week and showed partial improvement at 2 weeks post-SAH. Quantification of SI in the basis frontalis (Figure 4B) showed a significant delay and reduction in SI within hours after SAH. This reduction persisted for 1 week and began to recover 2 weeks post-SAH. The data indicated that the glymphatic function in the basis frontalis was severely impaired shortly after SAH but showed some recovery over time.

In the coronal-plane MRI images (Figure 4C), similar trends were observed. Before SAH (pre-SAH), Gd-DTPA penetration into the brain parenchyma was visible. One hour after SAH, there was a noticeable reduction in Gd-DTPA distribution in the PVS and brain parenchyma. The spread of Gd-DTPA, from the early influx to the late efflux, compromised significantly within 12 h post-injection (Figure S2B, D and E). Like the sagittal section, the impairment lasted for 1 week and showed partial recovery at 2 weeks post-SAH. Quantification of SI in the bilateral temporal bases (Figures 4D-E) showed a significant reduction in both the ipsilateral and contralateral hemispheres after SAH onset. The reduction was more severe in the ipsilateral hemisphere during the acute stage. This impairment persisted for 1 week and began to improve at 2 weeks post-SAH, with no significant difference between the hemispheres at this point (Figures 4C-E). Overall, these findings indicate that SAH induced severe and long-lasting impairment of the glymphatic system, particularly affecting the ipsilateral hemisphere more during the acute phase. Partial recovery of glymphatic function was observed at 2 weeks post-SAH.

### The meningeal lymphatic system for CSF clearance under normal conditions in beagles

**Figure 5:**
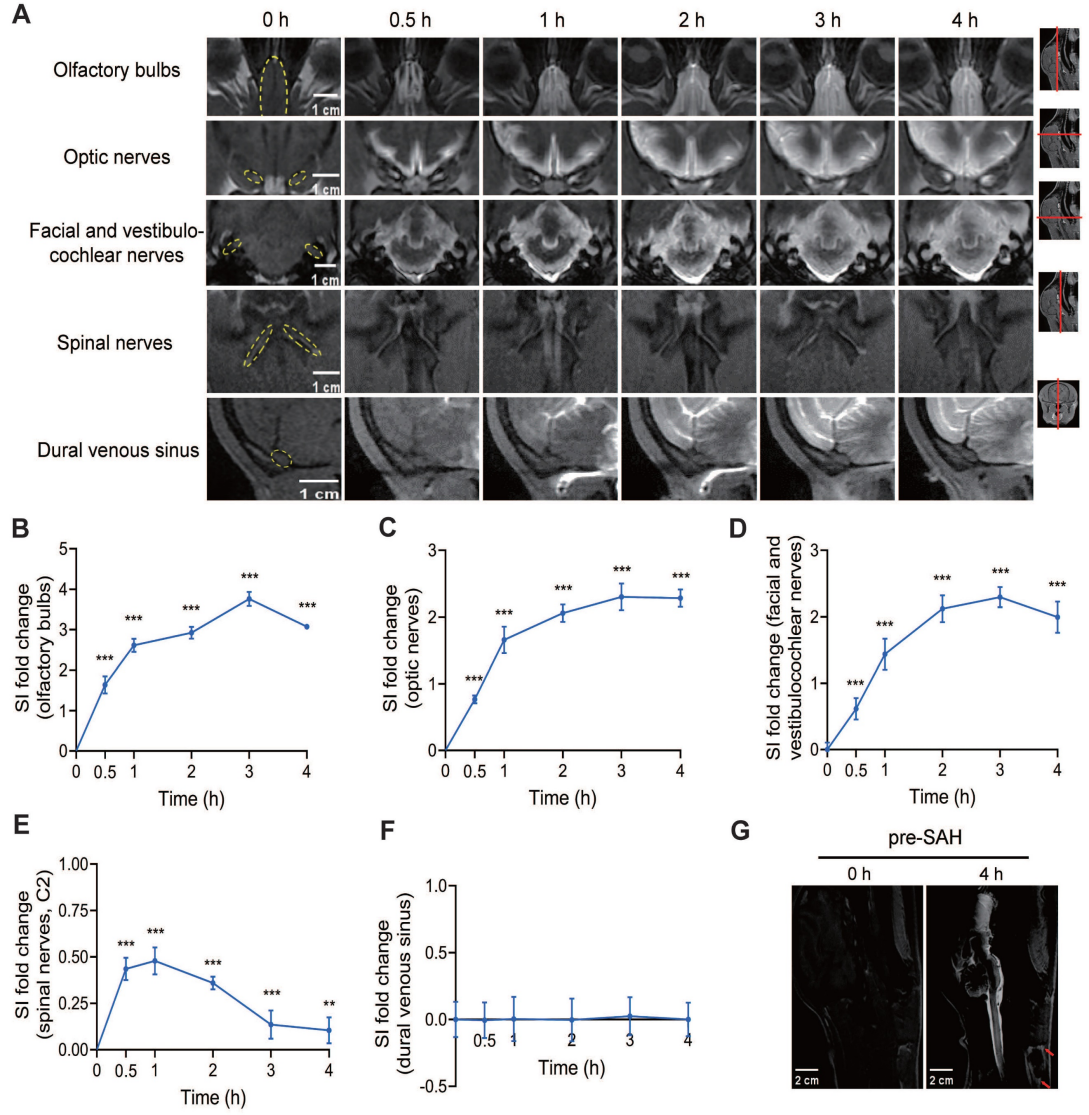
** Lymphatic clearance routes of CSF in pre-SAH beagles.** (A) Representative MRI images showing the outflow of Gd-DTPA along the olfactory bulbs, optic nerves, facial and vestibulocochlear nerves, spinal nerves (C2), and dural venous sinus at different time points after Gd-DTPA injection. (B to F) Quantification of SI fold changes over time for the olfactory bulbs (B), optic nerves (C), facial and vestibulocochlear nerves (D), spinal nerves (C2) (E), and the dural venous sinus (F), respectively, in pre-SAH beagles. (G) Representative MRI images showing enhanced cervical lymph nodes (red arrows) 4 h after Gd-DTPA injection. One-way ANOVA, n = 9; ** P < 0.01, *** P < 0.001 compared to 0 h. MRI: magnetic resonance imaging; SI: signal intensity.

The lymphatic clearance routes of CSF can be visualized by the contrast agent signal around several cranial and spinal nerves. Within the first 30 min after Gd-DTPA injection, a quick filling of the olfactory bulbs, optic nerves, facial and vestibulocochlear nerves, and spinal nerves was observed, indicating that the contrast agent rapidly reached these areas (Figure 5A). In contrast, the dural venous sinus did not exhibit significant quick filling, suggesting a limited role in immediate CSF clearance.

Analysis of SI showed that the outflow of Gd-DTPA via the olfactory bulbs, optic nerves, and facial and vestibulocochlear nerves increased over time, peaking at 3 h after injection and remaining high until 4 h (Figures 5B-D). After 4 h, the outflow of Gd-DTPA via these routes gradually decreased and persisted to 12 h after injection (Figure S3A-F). Although the spinal nerves also showed quick filling within the first 30 min, the SI values were much lower and exhibited a shorter rise compared to the cranial nerves, indicating a less prominent role in sustained lymphatic clearance (Figure 5E). The SI of the dural venous sinus showed no significant change after Gd-DTPA injection, further confirming its limited involvement in CSF clearance (Figure 5F).

Importantly, outside the skull, an obvious signal of Gd-DTPA was detected in the cervical lymph nodes 4 h after injection, indicating the successful drainage of CSF from the cranial and spinal nerves to the peripheral lymphatic system (Figure 5G).

### SAH impaired lymphatic clearance in beagles

**Figure 6:**
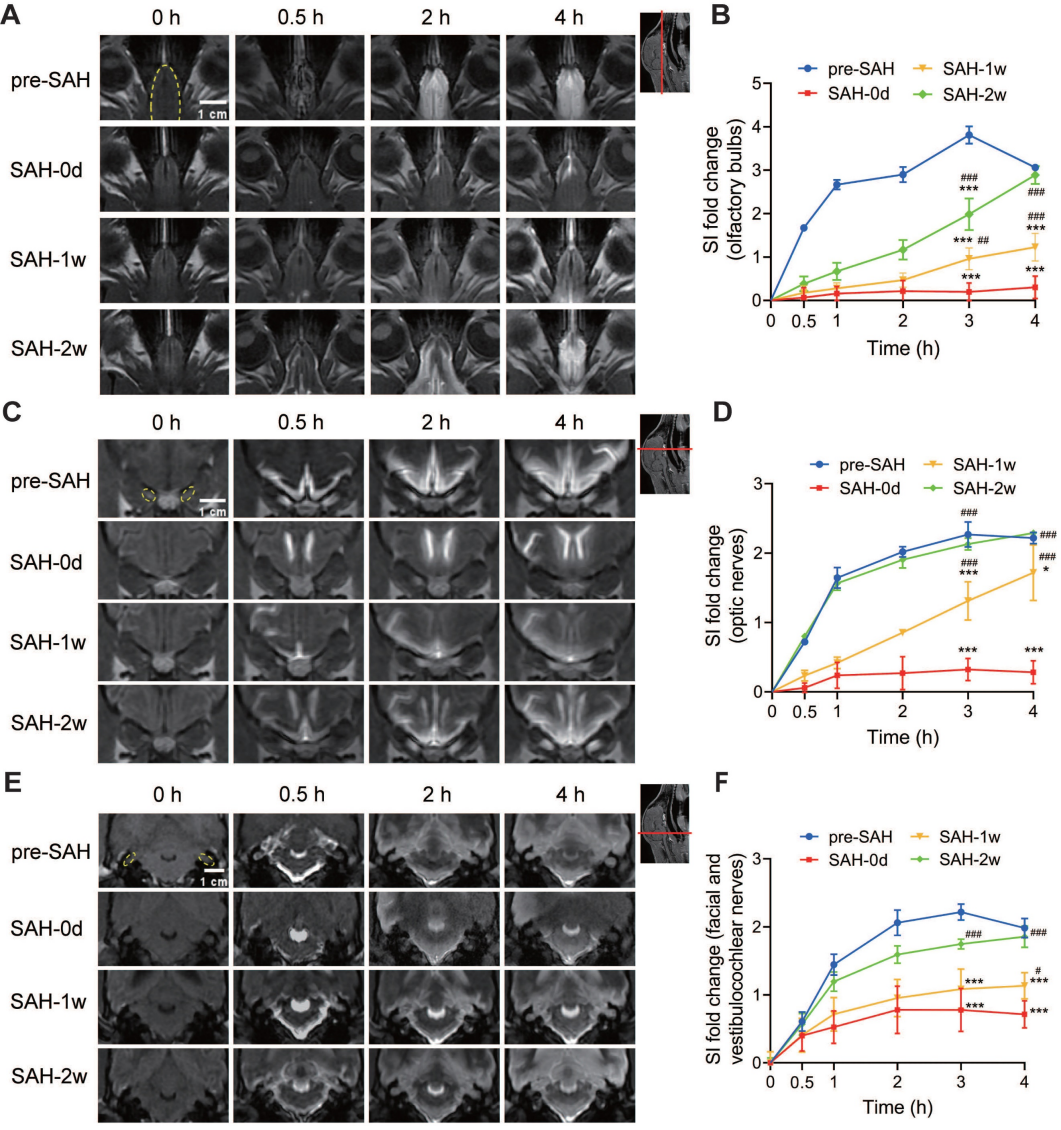
** SAH reduced the outflow of CSF through the olfactory bulbs, optic nerves, and the facial and vestibulocochlear nerves.** (A-B) SAH reduced the outflow of CSF through the olfactory bulbs. (A) Representative transverse-plane MRI images showing the outflow of Gd-DTPA via the olfactory bulbs before SAH (pre-SAH, 1st row), 1 h after SAH (SAH-0d, 2nd row), 1 week after SAH (SAH-1w, 3rd row), and 2 weeks after SAH (SAH-2w, 4th row). (B) Quantitative results showing that the drainage of Gd-DTPA via the olfactory bulbs significantly decreased within hours after SAH and almost recovered at 2 weeks. (C-D) SAH reduced the outflow of CSF through the optic nerves. (C) Representative coronal-plane MRI images showing the outflow of Gd-DTPA via the optic nerves before SAH (pre-SAH, 1st row), 1 h after SAH (SAH-0d, 2nd row), 1 week after SAH (SAH-1w, 3rd row), and 2 weeks after SAH (SAH-2w, 4th row). (D) Quantitative results showing that the drainage of Gd-DTPA via the optic nerves significantly decreased within hours after SAH, gradually recovered at 1 week, and returned to normal at 2 weeks. (E-F) SAH reduced the outflow of CSF through the facial and vestibulocochlear nerves. (E) Representative coronal-plane MRI images showing the outflow of Gd-DTPA via the facial and vestibulocochlear nerves before SAH (pre-SAH, 1st row), 1 h after SAH (SAH-0d, 2nd row), 1 week after SAH (SAH-1w, 3rd row), and 2 weeks after SAH (SAH-2w, 4th row). (F) Quantitative results showing that the drainage of Gd-DTPA via the facial and vestibulocochlear nerves significantly decreased within hours after SAH and recovered at 2 weeks. One-way ANOVA, n = 3 or 6; * P < 0.05, *** P < 0.001 compared to pre-SAH; # P < 0.05, ## P < 0.01, ### P < 0.001 compared to SAH-0d. CSF: cerebrospinal fluid; MRI: magnetic resonance imaging; SAH: subarachnoid hemorrhage.

For the lymphatic system, we observed a long-lasting delayed and reduced efflux of Gd-DTPA from the cisterna magna to the olfactory bulb (Figure 6A and Figure S3A), the optic nerves (Figure 6C and Figure S3B), and the facial and vestibulocochlear nerves (Figure 6E and Figure S3C) within the first few hours after SAH. In contrast, there was no significant change in the outflow via the spinal nerves (Figure S4A) and the dural venous sinus (Figure S4C). Quantification of SI in these regions for the lymphatic system showed a notable reduction in the olfactory bulb (Figure 6B and Figure S3D), the optic nerves (Figure 6D and Figure S3E), and the facial and vestibulocochlear nerves (Figure 6F and Figure S3F) within 12 h after Gd-DTPA injection compared to pre-SAH levels. However, the SI in the spinal nerves (Figure S4B) and dural venous sinus (Figure S4D) remained unchanged. Additionally, a slight increase in SI was observed in the spinal SAS after SAH, which normalized by 1 week (Figure S5A-B). No signal of Gd-DTPA was observed in the cervical lymph nodes after SAH (Figure S6). These results suggested that CSF drainage through the meningeal lymphatic routes along the olfactory bulb, optic nerves, and facial and vestibulocochlear nerves was acutely disrupted following SAH (Figure 6). The impairments lasted for 1 week and nearly recovered by 2 weeks post-SAH (Figure 6).

**Figure 7:**
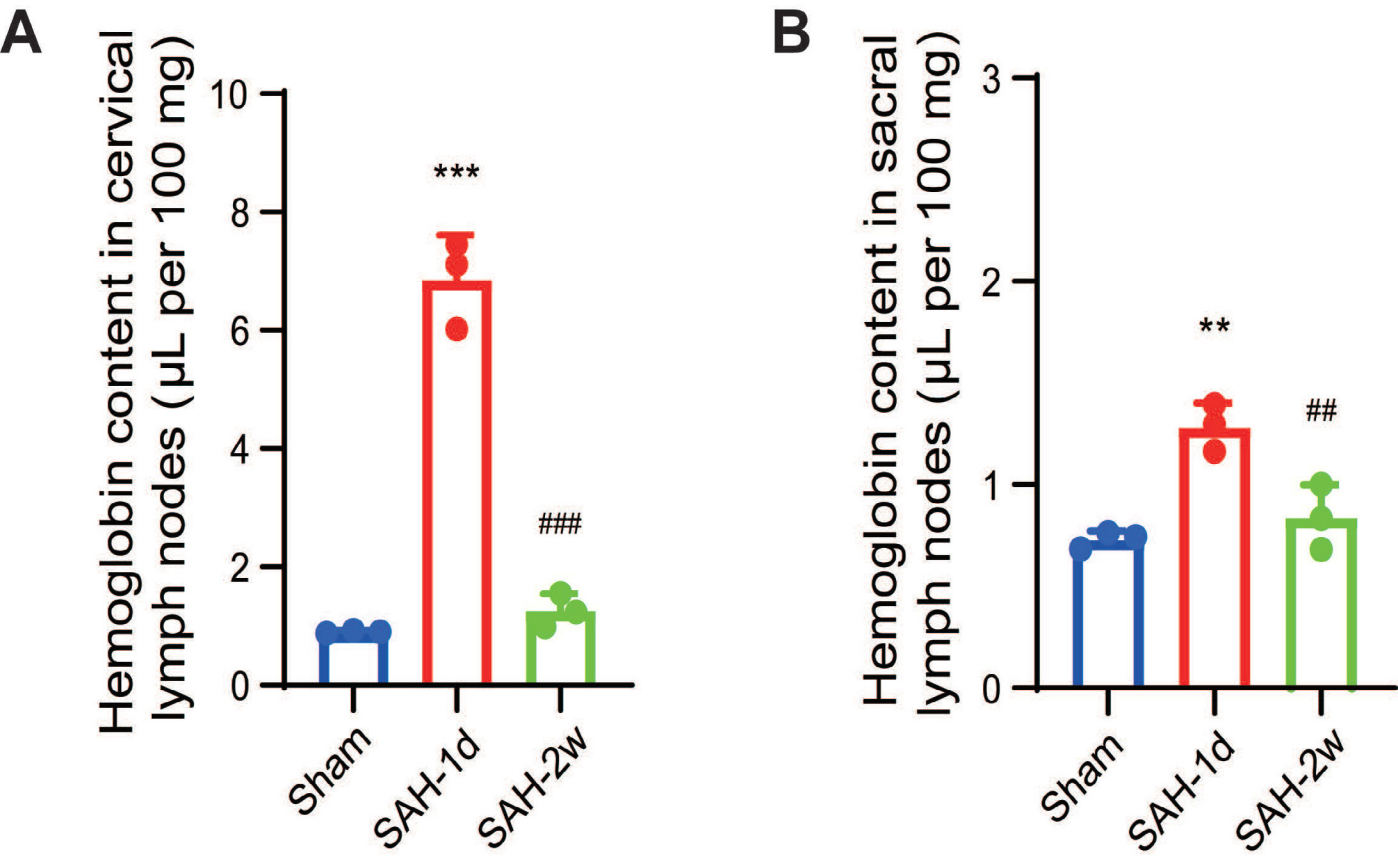
** SAH significantly increased hemoglobin content in both the cervical and sacral lymph nodes.** (A) Quantitative results showing hemoglobin content in the cervical lymph nodes. (B) Quantitative results showing hemoglobin content in the sacral lymph nodes. Hemoglobin content was measured using a colorimetric assay in isolated lymph nodes from Sham, SAH-1d, and SAH-2w beagles. One-way ANOVA, n = 3; ** P < 0.01, *** P < 0.001 compared to Sham; ## P < 0.01, ### P < 0.001 compared to SAH-1d. SAH: subarachnoid hemorrhage.

Despite the initial destruction of the lymphatic clearance pathways, we detected a significant increase in hemoglobin levels both in the cervical lymph nodes and the sacral lymph nodes 1 day after SAH (Figure 7A-B). This increase in hemoglobin levels indicated the presence of blood or blood components in the lymphatic system, suggesting that the meningeal lymphatic system was actively clearing the blood that had leaked into the CSF due to the hemorrhage. By 2 weeks, the hemoglobin levels had returned to normal, indicating that the lymphatic system had effectively cleared the hemorrhagic debris and was beginning to recover its normal function.

### Intermittent cistern magna CSF drain ameliorated the glymphatic and meningeal lymphatic function and improved the outcome after SAH

**Figure 8:**
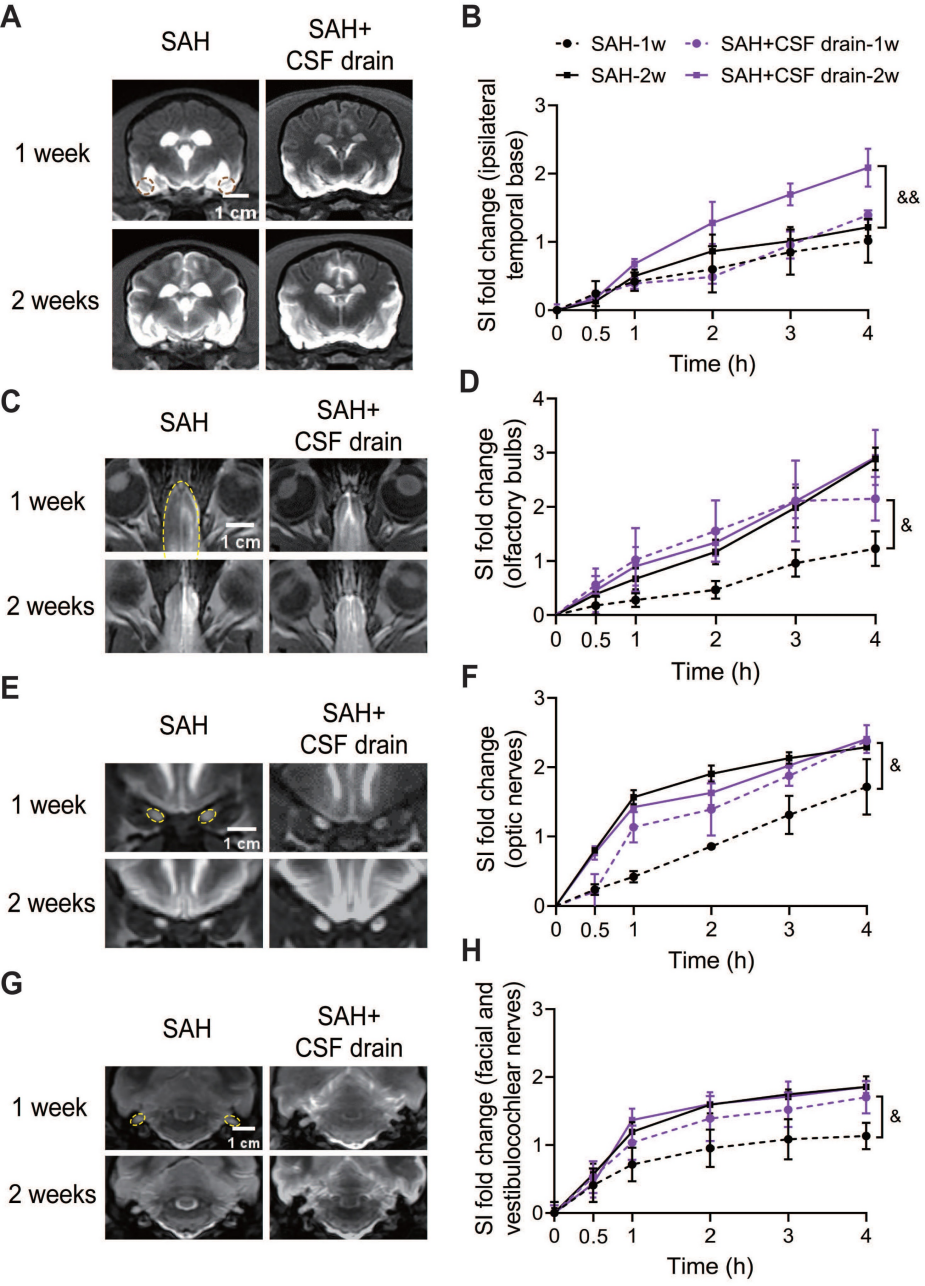
** Intermittent cistern magna CSF drain promoted the functional recovery of the glymphatic system and meningeal lymphatic system after SAH.** (A) Representative coronal-plane MRI images showing the distribution of Gd-DTPA in brain parenchyma 4 h after injection in SAH and SAH + CSF drain beagles 1 week (1st row) and 2 weeks after SAH (2nd row). (B) Quantification of signal intensity (SI) fold changes of Gd-DTPA over time in the ipsilateral temporal base. Intermittent cistern magna CSF drain significantly increased the signal intensity of Gd-DTPA in the ipsilateral temporal base 2 weeks after SAH. (C) Representative MRI images showing the outflow of Gd-DTPA along the olfactory bulbs (4 h after injection) in SAH and SAH + CSF drain beagles 1 week (1st row) and 2 weeks after SAH (2nd row). (D) Quantification of SI fold changes over time of the olfactory bulbs. (E) Representative MRI images showing the outflow of Gd-DTPA via optic nerves (4 h after injection) in SAH and SAH + CSF drain beagles 1 week (1st row) and 2 weeks after SAH (2nd row). (F) Quantification of SI fold changes over time of optic nerves. (G) Representative MRI images showing the outflow of Gd-DTPA via facial and vestibulocochlear nerves (4 h after injection) in SAH and SAH + CSF drain beagles 1 week (1st row) and 2 weeks after SAH (2nd row). (H) Quantification of SI fold changes over time of facial and vestibulocochlear nerves. Student *t* test, n = 3; & P < 0.05, && P < 0.01, compared to the SAH group. CSF: cerebrospinal fluid; SAH: subarachnoid hemorrhage; MRI: magnetic resonance imaging.

Intermittent cistern magna CSF drain significantly improved the glymphatic function, as shown by an increase of SI in basis frontalis (Figure S7A-B), the ipsilateral and contralateral temporal base (Figure 8A-B and Figure S7C) 2 weeks after SAH. No significant improvement in these areas was observed at 1 week (Figure S7A-B, Figure 8A-B, and Figure S7C). In the meningeal lymphatic system, an earlier recovery was found at 1 week after SAH in the olfactory bulbs (Figure 8C-D), optic nerves (Figure 8E-F) and facial and vestibulocochlear nerves (Figure 8G-H) after intermittent cistern magna CSF drain. In addition, much less stagnation of contrast media was visualized in the ventricles of the external CSF drain treated group (Figure 8A), demonstrating better CSF circulation. Intermittent cistern magna CSF drain for 3 days had no effect on the CSF pressure (Figure S7D).

**Figure 9:**
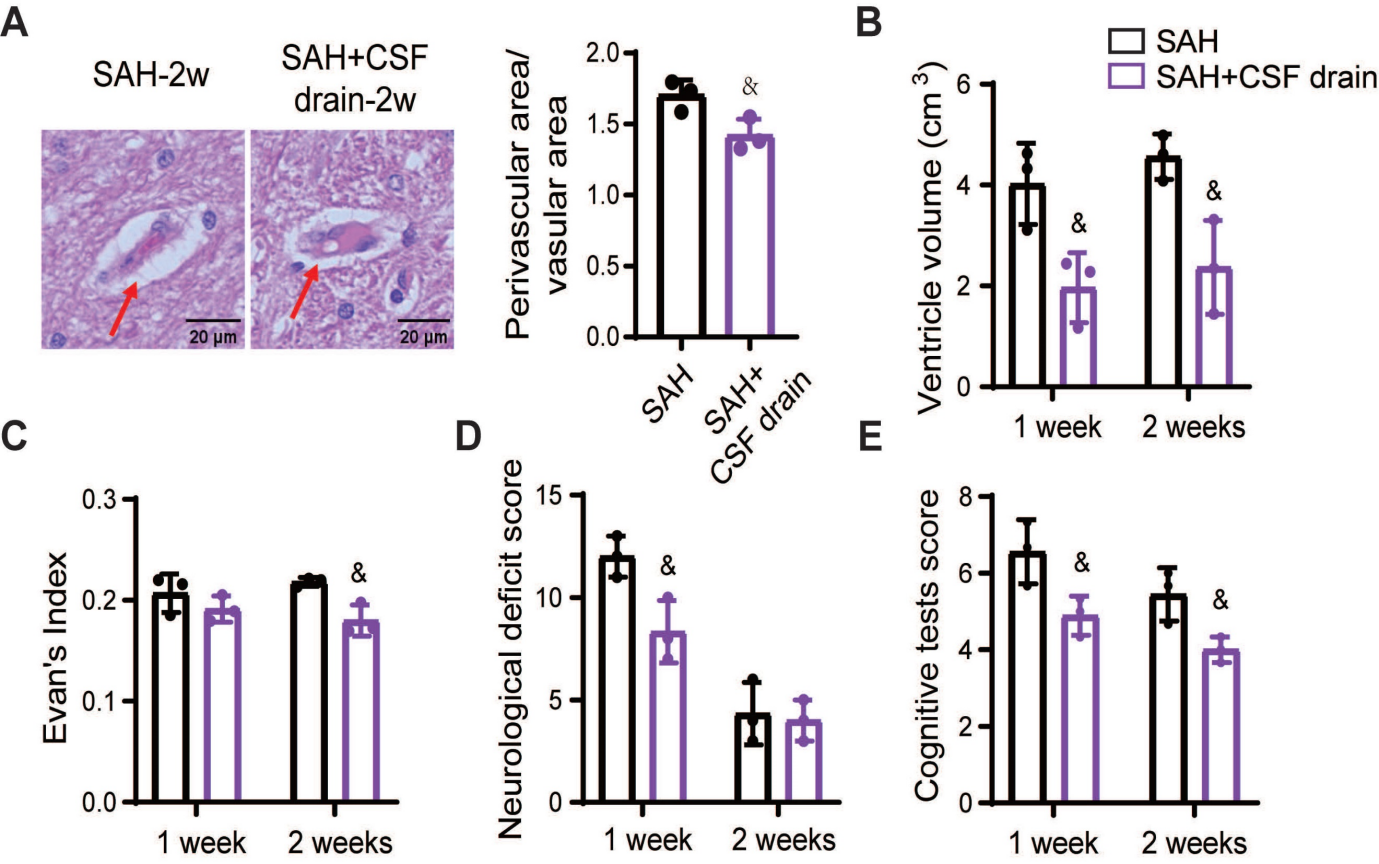
** Intermittent cistern magna CSF drain alleviated PVS enlargement, hydrocephalus, and neurological and cognitive deficits in SAH beagles.** (A) Left, representative hematoxylin-eosin staining showing the PVS 2 weeks after SAH with or without intermittent cistern magna CSF drain treatment; Right, quantification of the Left images. (B) Quantification of the lateral ventricle volume in SAH and SAH + CSF drain beagles 1 week and 2 weeks after SAH. Intermittent cistern magna CSF drain decreased the lateral ventricle volume in SAH beagles. (C) Quantification of Evan's index in SAH and SAH + CSF drain beagles 1 week and 2 weeks after SAH. (D) Quantification of the Neurological Deficit Scores in SAH and SAH + CSF drain beagles 1 week and 2 weeks after SAH. (E) Quantification of the cognitive test scores in SAH and SAH + CSF drain beagles 1 week and 2 weeks after SAH. Student *t* test, n = 3; & P < 0.05, compared to the SAH group. CSF: cerebrospinal fluid; PVS: perivascular space; SAH: subarachnoid hemorrhage.

To determine whether the benefits of intermittent cistern magna CSF drain on the glymphatic and meningeal lymphatic systems lead to advantageous outcomes in SAH beagles, we quantified the PVS, ventricle volume, and neurological and cognitive deficits. Intermittent cistern magna CSF drain alleviated the SAH-induced enlargement of PVS, showing a decreased ratio between perivascular and vascular areas (Figure 9A). Hydrocephalus was alleviated after treatment with external CSF drain, demonstrated by smaller ventricular volume (Figure 9B) and decreased Evan's index (Figure 9C). Accordingly, intermittent cistern magna CSF drain significantly improved post-SAH neurological deficits (Figure 9D) and cognitive impairment (Figure 9E). Collectively, these results suggested that external CSF drain is an effective therapeutic approach to facilitate the recovery of glymphatic and meningeal lymphatic function following SAH, contributing to the outcome improvement.

## Discussion

During the past decade, the importance of the glymphatic and meningeal lymphatic in brain waste clearance has been extensively investigated using intravenous or intracisternal injection of various fluorescence tracers or contrast agents in rodents 4,6,7,23. Few have been conducted on large animals. The dysfunction of the glymphatic and meningeal lymphatic system has been emphasized and implicated with brain damage in Alzheimer's disease, Parkinson's disease, and stroke 17,24-28. However, there is no systematic evaluation of the glymphatic and meningeal lymphatic dysfunction following SAH and their contribution to the brain pathophysiology.

Here, we successfully established the SAH model in beagles via endovascular perforation with a digital subtraction angiography machine. We chose the endovascular perforation model to simulate aneurysmal rupture, which most resembles the human SAH compared to other models. Comparing to the rodent lissencephalic brain, the canine brain has more similarities to humans in neuroanatomy and physiology, including well-defined sulci and gyri, lobulated cortexes and well-developed meninges 29. These allow a much closer representation of SAH patients. By using this novel large animal model, our study provided valuable insights into the dynamics of the glymphatic and meningeal lymphatic systems following SAH, and their association with the brain damage.

In this study, we did not use clinical scores like the Fisher scale but instead employed the Sugawara SAH grading system, originally developed for rats. The modified Fisher scale, while commonly used in SAH patients, has only moderate interrater reliability and is not validated for use in beagles 30. With no established SAH grading scale for beagles, we opted for the Sugawara system, which is widely used in preclinical models, including rodents and rabbits, and has a strong correlation with neurological deficits and cerebral vasospasm 22,31. We believe this system is more appropriate for assessing SAH severity in beagles.

### SAH caused irreversible impairment of the glymphatic system

In our study, following cisterna magna injection, Gd-DTPA distribution was characterized by diffusion from the brain surface into the parenchyma along periarteriolar spaces, suggesting the glymphatic system as a key CSF circulation pathway in beagles. A similar pattern was observed in idiopathic normal pressure hydrocephalus patients 32. Stomata in the pia mater over major arteries provide pathways for CSF to enter PVS from basal cisterns 33,34, with PVS allowing CSF to flow into the parenchyma and out via perivenous spaces to the venous system or meningeal lymphatics, facilitating metabolic waste clearance 4.

SAH acutely impaired the early influx and the late efflux of the glymphatic system in beagles, with a sharp decline in CSF influx to PVS and efflux from parenchyma within the first few hours, persisting for 2 weeks. This was evidenced by glymphatic dysfunction and enlarged PVS reducing waste clearance post-SAH 17,35,36. Three mechanisms underlie this: 1) Blood clots in SAS extended to PVS, obstructing CSF flow 17; 2) Blood components activated immune cells, causing perivascular inflammation 37,38; 3) Increased osmotic concentration of CSF due to blood and cytokines, causing cerebral edema and elevated intracranial pressure 39. SAH also redistributed aquaporin-4 on astrocytes, impairing water transport 36. The impaired glymphatic system, along with the associated inflammatory milieu, contributes significantly to vasospasm of penetrating arteries and increased intracranial pressure. These factors together exacerbate delayed cerebral ischemia following SAH, ultimately leading to neuronal death. Tissue plasminogen activator improved glymphatic function and neurological deficits by reducing fibrinogen deposition in PVS 17.

Two weeks post-SAH, CSF pressure normalized but glymphatic function only partially improved, with persistent PVS enlargement in the temporal cortex suggesting potential irreversibility. This might relate to increased blood-brain barrier permeability and persistent inflammation post-SAH. Luo *et al.* reported microglia and astrocytes activation expressing proinflammatory factors 7 days post-SAH 37. In addition, several studies have highlighted the profound impact of sleep disruption on glymphatic dysfunction 40,41. Considering the high prevalence of sleep disruption after SAH 42, it is likely that these disturbances further impair glymphatic clearance post-SAH.

Enlarged PVS led to fluid drainage stagnation, restricted blood flow, hypoperfusion, and hypoxia, associated with hydrocephalus and cognitive deficits 43,44. Our findings align, showing enlarged ventricles on MRI, with lasting neurological and cognitive impairments. Exacerbated PVS enlargement and irreversible glymphatic dysfunction may significantly contribute to long-term brain damage post-SAH.

### SAH caused acute dysfunction of the meningeal lymphatic system which recovered at 2 weeks

In our study, the major lymphatic CSF clearance routes in beagles included pathways alongside the perineurium of the olfactory bulbs, optic nerves, and facial and vestibulocochlear nerves, eventually arriving at the cervical lymph nodes. Traditionally, CSF clearance was thought to occur through arachnoid granulations and villi into venous sinuses. However, evidence shows CSF outflow predominantly through basal meningeal lymphatics along cranial/spinal nerves to peripheral lymph nodes 23,45. CSF from the brain flows through cranial nerve sheaths to cervical lymph nodes 23, while CSF from the spinal cord flows along spinal nerve roots to sacral and iliac lymph nodes 46. Our findings align with these studies, showing active cranial lymphatic routes compared to the dural venous sinuses and spinal pathways in beagles, consistent with mouse studies 23,47. Among cranial routes, the olfactory nerve is dominant for CSF drainage into cervical lymph nodes in mice and canines 47-49. Our results resonated with this, showing rapid Gd-DTPA arrival at the olfactory bulb, maintaining high intensity 4 h post-injection. Recent MRI studies indicate similar olfactory pathways for human CSF outflow 50.

SAH rapidly impaired lymphatic clearance routes, with signals in the olfactory bulb, optic nerves, and facial and vestibulocochlear nerves nearly invisible within hours in beagles. Reduced lymphatic outflow to cervical nodes was observed, with significantly decreased SI in these areas and lower enhancement slopes in SAH beagles compared to pre-SAH. This may be due to lymphatic vessel occlusion by erythrocytes and blood clots, and increased outflow resistance from high CSF pressure and inflammation 36,51,52. Interestingly, spinal SAS signals increased post-cisternal infusion, suggesting blockage of PVS and perineural pathways, consistent with studies in glioma mice 53. Notably, lymphatic clearance dysfunction almost recovered 2 weeks post-SAH, coinciding with erythrocyte clearance and reduced CSF pressure. This suggests the meningeal lymphatic system plays a major role in clearing erythrocytes and blood clots post-SAH. Increased hemoglobin content in cervical lymph nodes indicated significant erythrocyte drainage through meningeal lymphatics, aligning with reports on tau clearance 54. The glymphatic and lymphatic systems work together for CSF clearance 12,54. Impaired lymphatic function can overburden PVS and disrupt glymphatic clearance. Early restoration of lymphatic clearance may improve glymphatic function and mitigate brain damage post-SAH.

### Intermittent cistern magna CSF drain facilitate the recovery of glymphatic and meningeal lymphatic function following SAH

SAH causes acute increased intracranial pressure and hydrocephalus due to the mass effect from hemorrhage and CSF outflow obstruction. In clinic, external CSF drainage, such as placement of an external ventricular drain or lumbar drain, is usually applied to lower the intracranial pressure and help the clearance of blood clots in SAH patients 55,56. Several studies have reported that external CSF drainage is an effective strategy to clear the subarachnoid blood and enhance cerebral perfusion, leading to the improvement of delayed cerebral ischemia and long-term cognitive outcomes post SAH 57,58. However, the mechanism remains unknown. In Beagles, insertion of an external ventricular drain or lumbar drain is easy to cause drain malposition and drain dislodgement due to their spontaneous movement, and lumbar puncture is technically challenging. Therefore, we alternatively performed cistern magna puncture to drain CSF intermittently. As expected, intermittent cistern magna CSF drain for 3 days significantly prevented the development of hydrocephalus and improved neurological and cognitive deficits compared with the untreated group, which might be associated with the accelerated recovery of glymphatic and meningeal lymphatic function.

### Limitations

A major limitation of this study is that MRI scanning was performed under anesthesia, and the influence of anesthesia on CSF clearance should not be disregarded. Several studies have reported that anesthesia affects the dynamics of the glymphatic system and CSF drainage 59,60. For instance, Ketamine/Xylazine has been reported to increase glymphatic system activity in mice, potentially mediated by an increased vasomotor response through activation of α2-adrenergic receptors 60. Another limitation is the relatively small sample size, which is due in part to the high cost of beagles and the limited availability of MRI and DSA machines. Additionally, due to the lack of canine-specific antibodies, we were unable to demonstrate the presence of meningeal lymphatic vessels alongside existing cranial nerves using immunohistochemistry staining, as has been extensively demonstrated in mice.

## Conclusion

This study highlights the significant impact of SAH on the glymphatic and meningeal lymphatic systems in beagles. Our findings demonstrate that SAH acutely impairs glymphatic function, resulting in reduced CSF influx and enlarged perivascular spaces, which persist for up to 2 weeks. Additionally, the meningeal lymphatic system is rapidly disrupted following SAH, leading to diminished lymphatic outflow and increased hemoglobin content in cervical lymph nodes. The meningeal lymphatic system showed better recovery at 2 weeks, with improved CSF outflow and reduced hemoglobin content in cervical lymph nodes. These insights underscore the interconnected roles of the glymphatic and meningeal lymphatic systems in CSF clearance and the broader implications for post-SAH brain damage and recovery. External CSF drainage might be an effective therapeutic approach to facilitate the recovery of glymphatic and meningeal lymphatic function following SAH.

## Supplementary Material

Supplementary figures.

## Abbreviations

ACA: anterior cerebral artery
BA: basal artery
CSF: cerebrospinal fluid
Gd-DTPA: dimeglumine gadopentetate
MRI: magnetic resonance imaging
NDS: Neurological Deficit Score
PBS: phosphate buffered saline
PCA: posterior cerebral artery
PVS: perivascular space
SAH: subarachnoid hemorrhage
SAS: subarachnoid space
SI: signal intensity
