# Supplementary material for: Subarachnoid hemorrhage distinctively disrupts the glymphatic and meningeal lymphatic systems in beagles
Source: Theranostics. 2024 Sep 16;14(15):6053–70. doi: 10.7150/thno.100982 (PMC11426235; doi:10.7150/thno.100982)
Supplement: Supplementary file 1 — Supplementary figures. [file thnov14p6053s1.pdf]

SUPPLEMENTARY MATERIAL

Subarachnoid hemorrhage distinctively disrupts the glymphatic and meningeal lymphatic system in beagles

Jiaqi Wang[1,2 #], Tao Lv[1 #], Feng Jia[1 #], Yang Li[3], Weiwei Ma[3], Zhi-Peng Xiao[4], Weifeng Yu[2], Heng Zhao[5*], Xiaohua Zhang[1*], Qin Hu[1, 2, 6 *]

[1] Department of Neurosurgery, Ren Ji Hospital, Shanghai Jiao Tong University School of Medicine, Shanghai, China.

[2] Key Laboratory of Anesthesiology (Shanghai Jiao Tong University), Ministry of Education, China.

[3] Department of Radiology, Ren Ji Hospital, Shanghai Jiao Tong University School of Medicine, Shanghai, China.

[4] Jiangxi Key Laboratory of Neurological Diseases, Department of Neurosurgery, The First Affiliated Hospital, Jiangxi Medical College, Nanchang University, Nanchang, Jiangxi, China.

[5] Beijing Institute of Brain Disorders, Laboratory of Brain Disorders, Ministry of Science and Technology, Joint Innovation Center for Brain Disorders, Capital Medical University, Beijing, China.

[6] Loma Linda University School of Medicine, Loma Linda, CA 92350, USA.

[#]These authors contributed equally.

[*]Corresponding authors.

Address for correspondence:

Qin Hu, Department of Neurosurgery, Ren Ji Hospital, Shanghai Jiao Tong University School of Medicine, Shanghai, China. Email: huqinle2019@sjtu.edu.cn

Xiaohua Zhang, Department of Neurosurgery, Ren Ji Hospital, Shanghai Jiao Tong University School of Medicine, Shanghai, China. Email: zhangxiaohua011517@renji.com

Heng Zhao, Beijing Institute of Brain Disorders, Capital Medical University, Beijing, China.

E-mail: heng.zhao10@gmail.com

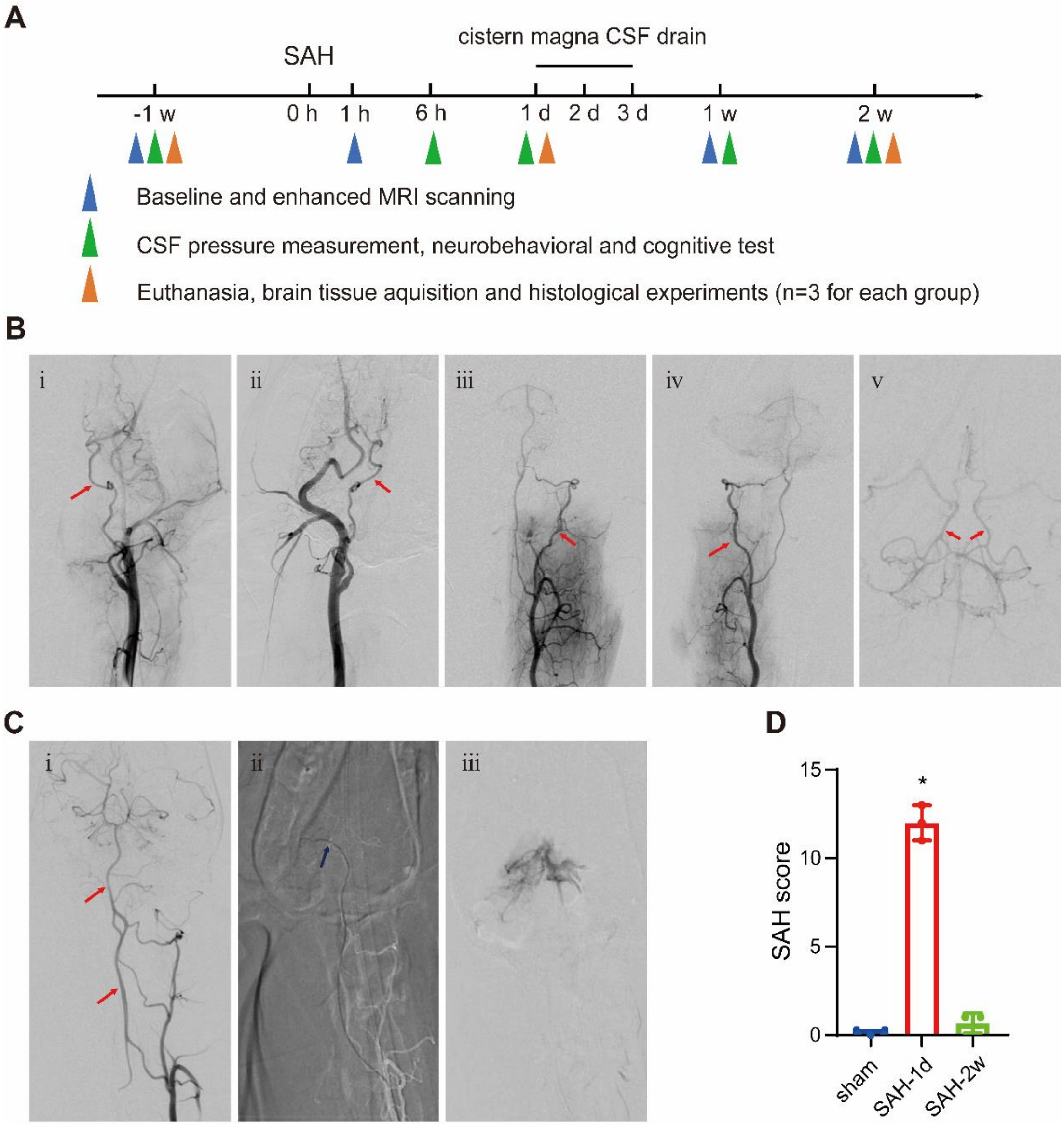

Figure S1. The experimental design and the establishment of SAH model in beagles. (A) The experimental design and timeline of SAH induction, MRI scan, CSF pressure measurement, neurological and cognitive tests, and histological study in beagles. (B) Representative images of beagle cerebral arteries (red arrows) on DSA. i) Left internal cerebral arteries; ii) Right internal cerebral arteries; iii) Left vertebral artery; iv) Right vertebral artery and v) the Circle of Willis. (C) Representative images showing the procedure of SAH induction. i) Posteroanterior view of the vessel passage to perform the guidewires and catheters towards the Circle of Willis (red arrows); ii) Advance the microguidewire into the Circle of Willis and puncture at the right posterior communicating artery (blue arrow); iii) Extravascular outflow of the contrast media demonstrated the success of SAH induction. (D) SAH significantly increased the SAH scores at 1 day, which returned to normal at 2 weeks. One-way ANOVA, n = 3; *$P < 0.05$ compared to sham. CSF: cerebrospinal fluid; DSA: digitalsubtraction angiography; MRI: magnetic resonance imaging; SAH: subarachnoid hemorrhage.

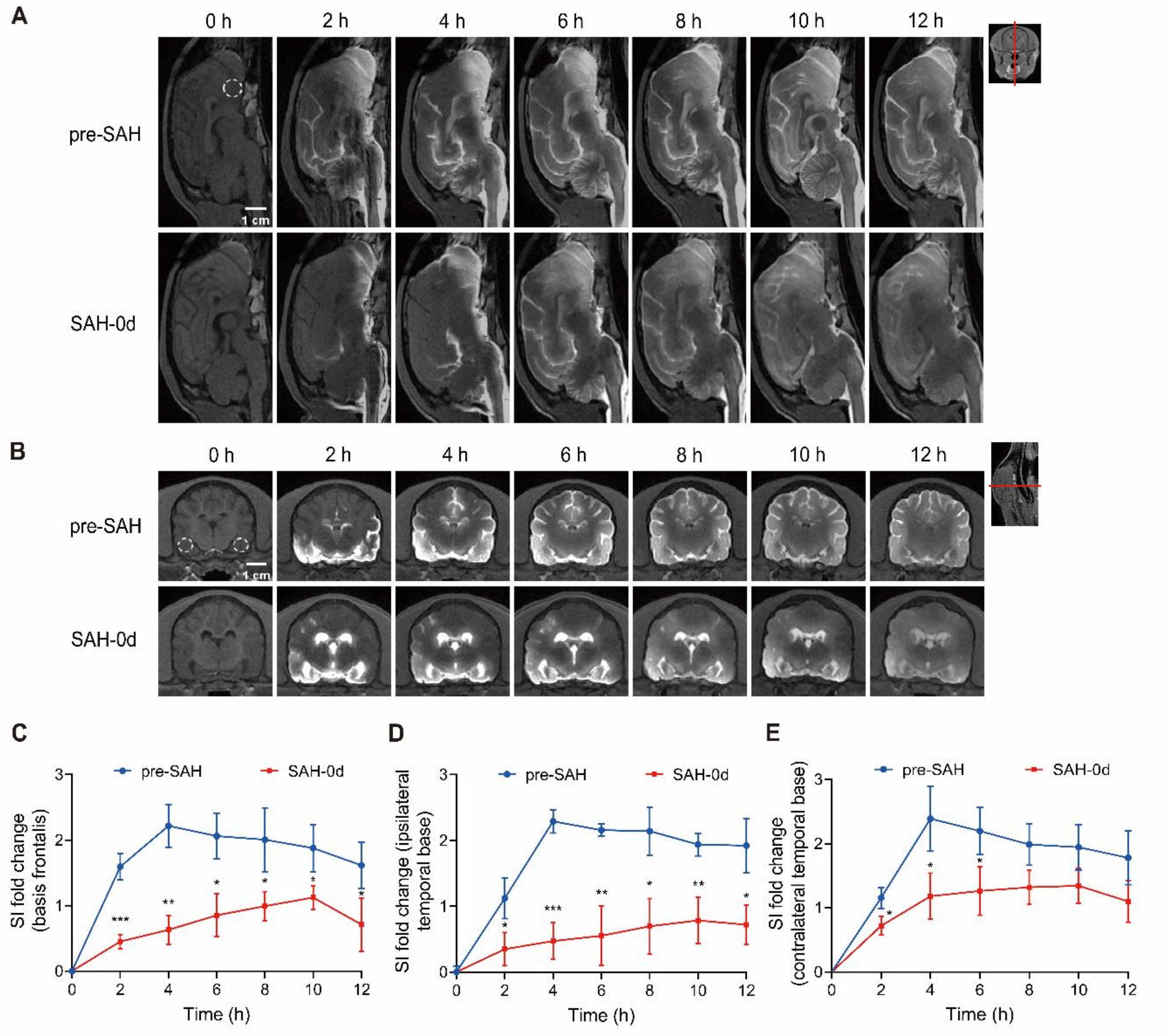

Figure S2. SAH impaired the early influx and late efflux of Gd-DTPA in the brain parenchyma. (A) Representative midsagittal-plane MRI images showing the accumulation and dissipation of Gd-DTPA from 2 h to 12 h after Gd-DTPA injection in beagles before (pre-SAH) and 1 h after SAH (SAH-0d). (B) Representative coronal-plane MRI images of (A). (C) Quantification of SI fold changes over time in the basis frontalis in (A). (D) Quantification of SI fold changes over time in the contralateral temporal base in (B). (E) Quantification of SI fold changes over time in the ipsilateral temporal base in (B). The accumulation of Gd-DTPA peaked at 4 h, began to dissipate at 6 h, and residual intensity was still observed 12 h after Gd-DTPA injection in pre-SAH beagles. SAH significantly disrupted the accumulation and dissipation of Gd-DTPA. Student *t* test, n = 3; * $P < 0.05$, ** $P < 0.01$, *** $P < 0.001$, compared to pre-SAH.SAH: subarachnoid hemorrhage; MRI: magnetic resonance imaging.

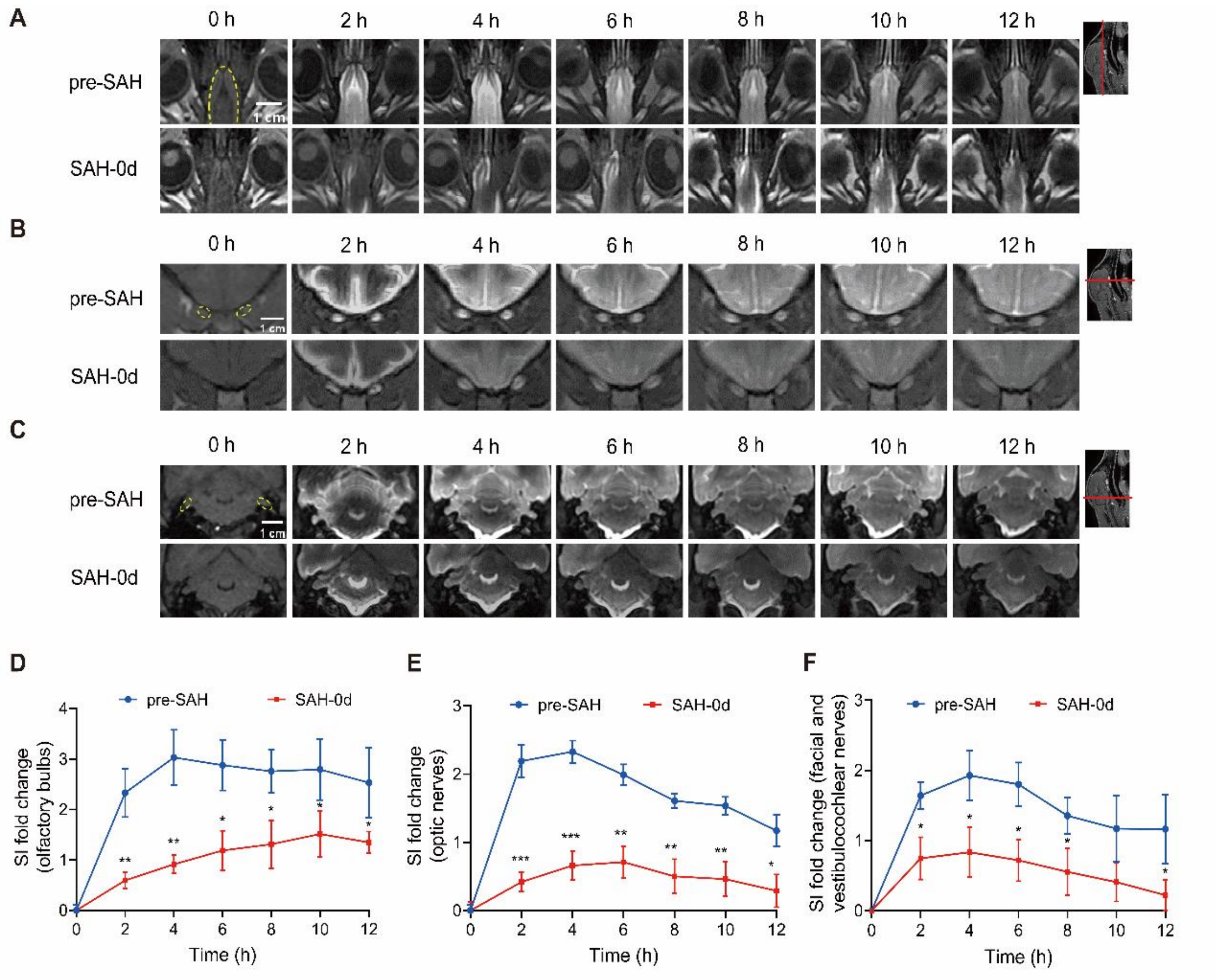

Figure S3. SAH impaired the outflow of Gd-DTPA in meningeal lymphatics along olfactory bulbs, optic nerves, facial and vestibulocochlear nerves. (A-C) Representative MRI images showing the outflow of Gd-DTPA along olfactory bulbs (A), optic nerves (B), and facial and vestibulocochlear nerves (C) from 2 h to 12 h after Gd-DTPA injection in beagles before and 1 h after SAH (SAH-0d). (D-F) Quantification of SI fold changes over time for olfactory bulbs (D), optic nerves (E), and facial and vestibulocochlear nerves (F). SAH significantly reduced the outflow of Gd-DTPA in these routes. Student *t* test, n = 3; * $P < 0.05$,** $P < 0.01$, *** $P < 0.001$, compared to pre-SAH. SAH: subarachnoid hemorrhage; MRI:magnetic resonance imaging.

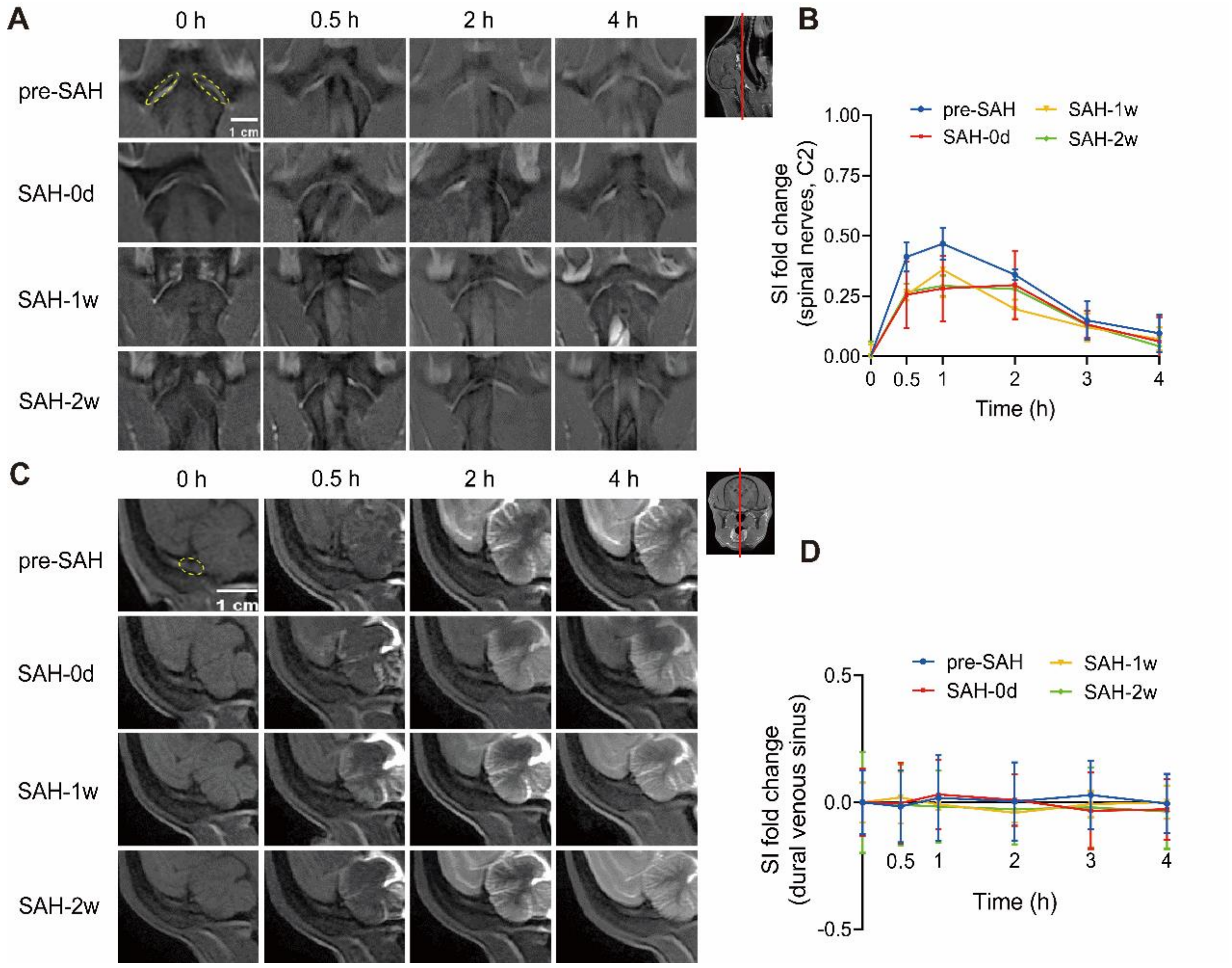

Figure S4. SAH had no effect on the outflow of Gd-DTPA through spinal nerves and dural venous sinus. (A-B) SAH didn't affect the outflow of Gd-DTPA through spinal nerves. (A) Representative transverse-plane MRI images showing the outflow of Gd-DTPA via spinal nerves before SAH (pre-SAH, 1st row), 1 h after SAH (SAH-0d, 2nd row), 1 week after SAH (SAH-1w, 3rd row), and 2 weeks after SAH (SAH-2w, 4th row). (B) Quantitative results showing that there was no significant difference in the outflow of Gd-DTPA via spinal nerves between pre- and post-SAH. (C-D) SAH had no effect on the outflow of Gd-DTPA through dural venous sinus. (C) Representative sagittal-plane MRI images showing the presence of Gd-DTPA in dural venous sinus before (pre-SAH, 1st row), 1 h after SAH (SAH-0d, 2nd row), 1 week after SAH (SAH-1w, 3rd row), and 2 weeks after SAH (SAH-2w, 4th row). (D) Quantitative results showing that there was no significant difference in the outflow of Gd-DTPA via dural venous sinus between pre- and post-SAH. One-way ANOVA, n = 3 or 6. SAH:subarachnoid hemorrhage.

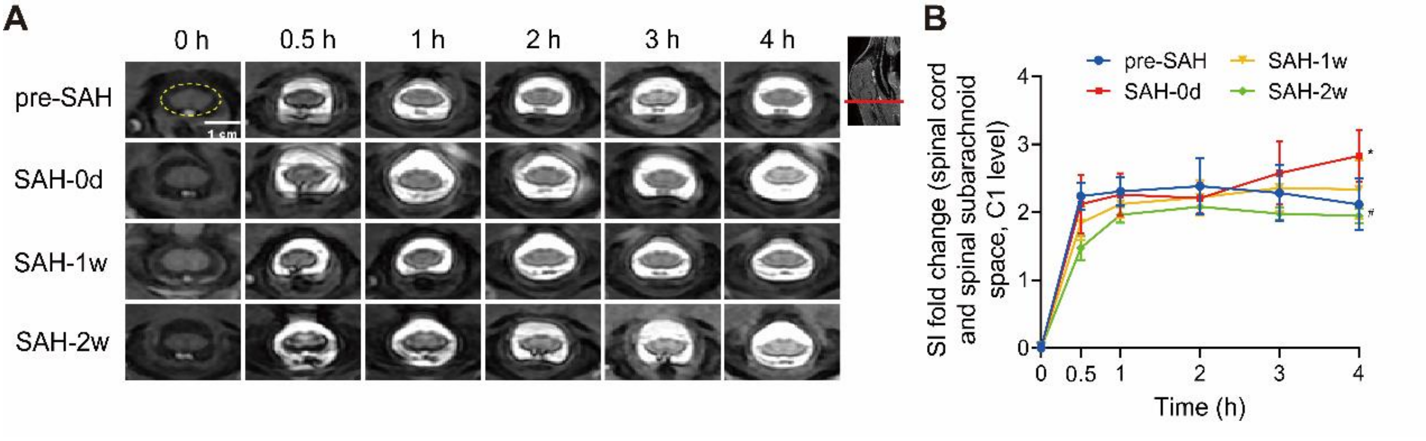

Figure S5. SAH slightly increased the retention of Gd-DTPA in spinal SAS. (A) Representative coronal-plane MRI images showing the presence of Gd-DTPA in spinal SAS before (pre-SAH, 1st row), 1 h after SAH (SAH-0d, 2nd row), 1 week after SAH (SAH-1w, 3rd row), and 2 weeks after SAH (SAH-2w, 4th row). (B) Quantitative results showing that SAH slightly increased the retention of Gd-DTPA in spinal SAS within hours and returned to normal level at 1 week. One-way ANOVA, n = 3 or 6. * $P < 0.05$ compared to pre-SAH; # $P < 0.05$ compared with SAH-0d. SAS: subarachnoid space, SAH: subarachnoid hemorrhage.

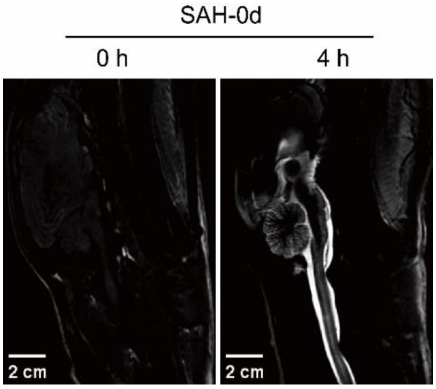

Figure S6. No visible diffusion of Gd-DTPA was observed at the cervical lymph nodes 4 h after injection in SAH beagles. SAH: subarachnoid hemorrhage.

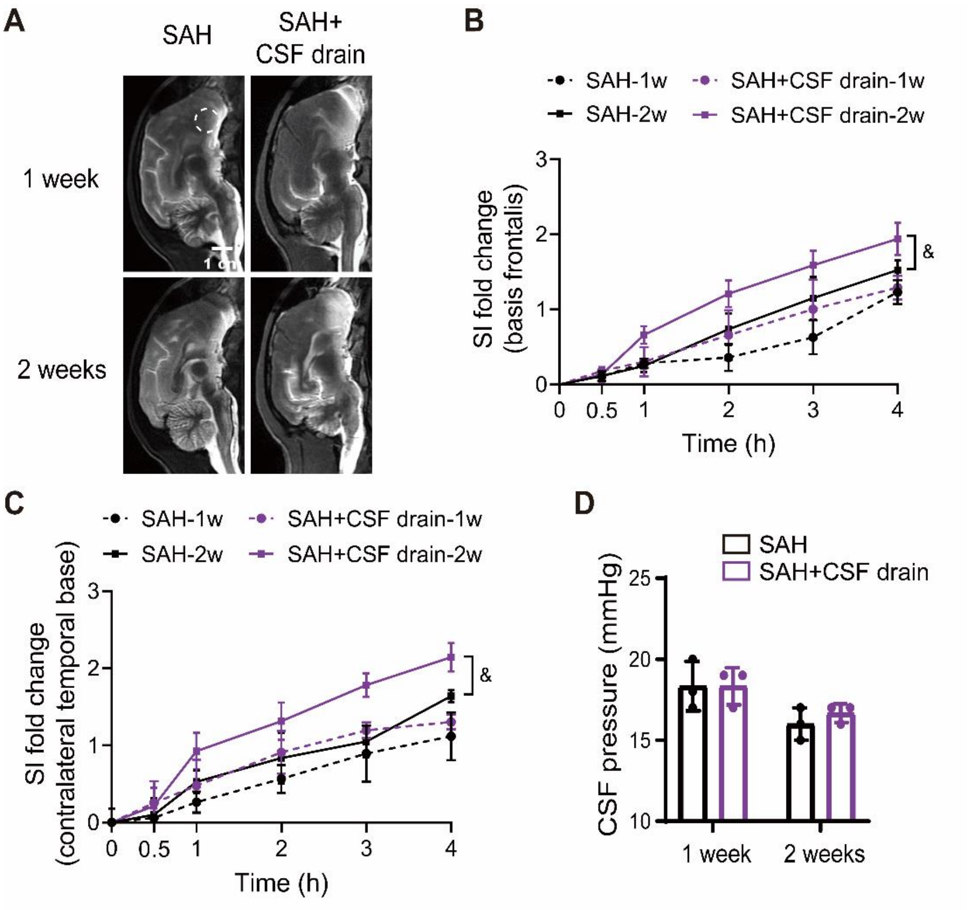

Figure S7. Intermittent cistern magna CSF drain promoted the recovery of the glymphatic function. (A) Representative midsagittal-plane MRI images showing Gd-DTPA parenchymal penetration (4 h) 1 week (1st row) and 2 weeks (2nd row) after SAH with or without CSF drain. (B) Quantification of SI fold changes over time in the basis frontalis of (A). Intermittent cistern magna CSF drain increased the accumulation of Gd-DTPA at basis frontalis 2 weeks after SAH. (C) Quantification of SI fold changes over time at the contralateral temporal base 1 week and 2 weeks after SAH (Figure 8A). Intermittent cistern magna CSF drain increased the accumulation of Gd-DTPA at the contralateral temporal base 2 weeks after SAH. (D) Intermittent cistern magna CSF drain had no effect on the CSF pressure in beagles 1 week and 2 weeks after SAH. Student *t* test, n = 3; & $P < 0.05$ comparedto SAH group. CSF: cerebrospinal fluid; MRI: magnetic resonance imaging; SAH: subarachnoid hemorrhage.
